# A History of the Improvements Which Practical Medicine Has Derived from Auscultation; Being the Essay to Which the Medical Society of Bordeaux Awarded Its Prize in November, 1839

**Published:** 1842-08

**Authors:** G. Peyraud

**Affiliations:** Doctor of Medicine of the Faculty of Paris, Corresponding Member of the Bordeaux Medical Society


### WESTERN JOURNAL

OF

### MEDICINE AND SURGERY.

AUGUST, 1842.

ART. I.—*A History of the Improvements which Practical Medicine has derived from Auscultation; being the essay to which the Medical Society of Bordeaux awarded its prize in November*, 1839. By G. PEYRAUD, Doctor of Medicine of the Faculty of Paris, Corresponding Member of the Bordeaux Medical Society. *Quæ fundata sunt in natura, crescunt et perficiuntur; quæ vero in opinione, variantur, non augentur.*—Baglivi. Translated from the French for the Western Journal of Medicine and Surgery, by CHARLES A. POPE, M. D., of St. Louis, Lecturer on Anatomy and Surgery, Physician to the St. Louis Dispensary, &c., &c.

(*Concluded.*)

### CHAPTER IV.—OF DISEASES OF THE HEART.

Thus far my province has been to state the real progress which the science of diagnosis owes exclusively to auscultation; I have shown that this admirable discovery has rendered it as easy to acquire a knowledge of the diseases of the lungs and pleura, as it was formerly difficult. Here my office changes, and instead of an exclusive admiration of the results obtained, I shall be led to inquire whether positive results have been really established, and whether the pathology of the heart has gained any thing by auscultation. But before entering on the discussion of this question, which, I confess, may appear to many persons a true paradox, let us see where the science was, relative to diseases of the chest, when Laennec began to apply his new method of exploration to their study.

Of all the discoveries which physiology has ever made, the most brilliant, beyond contradiction, and that which has exerted the most influence on pathology, is the discovery of the circulation of the blood. Besides the new light which it shed on one of the most important functions of the economy, it also proved that the ancients did not know and say every thing. The magic which surrounded the great name of Galen once destroyed, physicians became more bold, and no longer restricting themselves to the sterile and ungrateful task of commentators, entered the vast field of observation, where such rich harvests awaited them. Of all parts of pathology, the one which was necessarily to gain most by this scientific revolution, was that relating to diseases of the heart, which organ Harvey had just shown to be clothed with functions so important, and up to his time unknown. Before this ever celebrated epoch in the fasti of the art, the study of these diseases had been completely neglected, and if some scattered observations are met with here and there in the authors anterior to the seventeenth century, it is only as rare, curious, and extraordinary facts that they are reported, and not at all with the view of their co-ordination to refer them to a theory. But when once Harvey had made known his beautiful discovery, in 1619, the animated discussion which arose on this question, led to important researches, all of which profited the pathology of the heart, by throwing more and more light upon its structure, and the mechanism of its functions. From that time Lower, Ruysch, Vieussens, Leuwenhoeck, and Haller, multiplied these researches and experiments so as to leave scarcely any thing more to be discovered in the anatomy and physiology of the heart; whilst Lancisi, Albertini, Bonnet and Valsalva successively enriched its pathological history with a multitude of curious facts, which it only remained for Morgagni to complete, reunite and compare, with his remarkable sagacity, to make of his seventeenth and eighteenth letters, which he has devoted to aneurisms of the heart and of the aorta, one of the most remarkable parts of his great work.

It does not enter into my plan to analyse the writings of these different authors, in which, not even excepting Morgagni, I would find many anatomical details, but very few data calculated to make the diagnosis plainer. I shall restrict myself to two writers whose works seem to me to sum up pretty well what has been best said on diseases of the heart, before Laennec: I mean Sénac and Corvisart.

The *Traité de la structure du cœur, de son action et de ses maladies*, by Sénac, published in 1749, is certainly the most remarkable which had appeared at that time on this important subject. This immense work is divided into six books, the last of which only is devoted to diseases of the heart. It is therefore the only one that I shall notice, whatever be the merit of the first five, in which the author treats at length of the anatomy and physiology of the heart, and their merit is so great that it required nothing less than the incomparable éclat of the works of Haller to eclipse them a little at the epoch when they appeared.

The idea that predominates in the first chapters which Sénac devotes to diseases of the heart, is the extreme difficulty of their diagnosis. After having shown that diseases foreign to the heart simulate the latter so closely as almost infallibly to lead into error, he adds: "But when by distinguishing between the symptoms of other parts and those which arise from the heart, we ascertain the latter to be diseased, new difficulties occur: where is the cause of the disorder? It may be in the ventricles, in the auricles, or in the various parts enclosed by them; this is all we know about it: we are ignorant whether it is in the right cavities or in the left; the symptoms, which ought to vary according to the parts which produce them, are not different; they reduce themselves to palpitations, trembling, inequalities, in a word to a deranged action." (Senac, op. cit., vol. ii, p. 315).

We see whether Sénac dissembled the difficulties of the question. Perhaps, indeed, this emphatic declaration was a condition of success, for the questions which are most deeply penetrated, are those which have been thus examined in all their phases. Our author therefore courageously undertakes the solution of the problem which he proposes, and we cannot but admire the sagacity he evinces in separating the uncertainties from it. He considers, in the first place, the type of the disease; and from its intermittent or continued character, from the permanency of the symptoms or their momentary cessation, he infers the existence of organic lesions or the healthy state of the heart. The pulse also furnishes him important signs: its constant smallness and irregularity denote an alteration of the auricles or ventricles; the contrary state proves that the heart is not altered in its organization. If the symptoms are developed after a violent passion, as anger, or after a strain or blows on the sides, some disease of the heart is to be feared; whilst, if this organ has been agitated from a slight cause only, for example, the suppression of the catamenia, it is probable that the nerves create the disorder in its movements. The duration of the disease is likewise an important circumstance: thus the reappearance of dropsies is a strong reason for suspecting a disease of the heart. Lastly, if it be impossible to recognise certain diseases in particular, they may be known in general, and, although it cannot be determined whether the valves be ossified or whether there be a tumor in the ventricles, still it is not a matter of indifference to know that the heart is diseased.

I cannot but confess that these elements alone do not suffice to enable us to arrive at a certain or even probable diagnosis of a lesion of the heart; but it is not Sénac's fault, and he deserves praise for having learned how to profit by them as much as he has: there was but one practitioner so enlightened who could thus artificially create a diagnosis from inductions whose basis is apparently so slight. Besides, when the occasion presents itself, he does not neglect to draw signs elsewhere than from these data. Thus, it was he who pointed out the fluctuation of the liquid between the fifth and sixth ribs, in certain cases of hydro-pericardium; a sign which does not occur so frequently as he supposed, but which Corvisart and M. Bouillaud have since met with.

I pass over the chapters which he devotes to inflammations of the heart, and to the study of various other lesions, such as vegetations, osseous or calcareous concretions of the valves, displacements of the heart, etc., chapters remarkable for the great number of facts which are found therein, and for the judicious criticism to which these facts are submitted, but which present nothing of interest as it respects diagnosis, which was as difficult, or rather, as impossible for Sénac, as it yet is for us; and I come to that in which he treats of aneurisms of the heart.

The signs on which he seeks to establish the diagnosis are the following: dull and heavy pains in the region of the heart; difficulty of respiration, which he attributes to the depression of the diaphragm by the dilated heart (the constraint of the lungs would have been a much more natural explanation); small, quick, and irregular pulse; universal palpitation of the whole arterial system, which is felt at the same time in the head, arms and thighs (Sénac grants indeed that this sign does not belong exclusively to aneurisms of the heart, and that he has observed it in other diseases of this organ); beating of the jugulars, swelling of the extremities, and sometimes effusion in the cavities. (Op. cit., vol. ii. p. 473, et seq.) Sénac had too much judgment not to perceive, that there was not one of these signs which did not equally belong to all the diseases of the heart; therefore he soon adds another which, he says, *is more than conjecture and probability:* "If the hand applied to the region of the heart, feels a mass which strikes the ribs in a great extent, it is a decisive proof of dilatation." (Op. cit., vol. ii, p. 485). But in cases of simple hypertrophy of the heart without dilatation of its cavities, the heart strikes the sternum with a strong and quick stroke, which may be taken for that of a mass, on account of the vibration which it communicates to the whole sternum. It is true, that Sénac did not yet distinguish hypertrophy from simple dilatation, a distinction which has not been well established until since Corvisart's time.

Farther on, endeavoring to establish a differential diagnosis between aneurisms of the heart and those of the aorta, he reduces the characteristic signs of the latter to three principal ones: 1. seat of the pulsations in some other point of the thorax than the præcordial region, and which varies according to the part of the aorta which is diseased; 2. pains much greater in aneurisms of the aorta, than in those of the heart; 3. production of exterior tumors by the first, which, when they are voluminous, cause absorption of the ribs and project without. (Op. cit., vol. ii, p. 489) Since Sénac, no new differential sign has been indicated, and it must be avowed that when they present themselves united, which unfortunately does not in all cases happen, they must certainly throw great light upon the diagnosis.

Here I terminate the analysis of the work of Sénac, and I think I may say, in conclusion, that it is the most complete one which had yet appeared on this subject, and that it would be still almost on a level with the science, but for the labors of Corvisart and of the modern anatomical school.

The work of Corvisart (*Essai sur les maladies et les lésions organiques du cœur et des gros vaisseaux*), is in the first place distinguished by the application of the method of Avenbrugger to the diagnosis of those diseases, which at once gives it a marked preeminence over every thing which had been published up to that time. This mode of exploration is so natural for affections of the heart, that I should not be loth to believe it was in devoting himself to their study that Avenbrugger discovered it. Where indeed shall we find a more striking contrast than that which exists between the dulness of the precordial region, and the sonorousness of the neighboring part of the chest? The limits within which these various phenomena take place, is always precise, and where the flatness ceases, the sonorousness commences, without there being an intermediate space where they are confounded. The reason is simple: the heart, a thick, massive organ, is immediately enveloped on all sides, except in front, by the lungs, which always contain air (in the healthy state, I mean), and consequently are always light and crepitant. The enlargement of the space where the precordial flatness is heard, becomes therefore an important sign, especially when combined with the other rational signs, of which Corvisart has given a more correct and profound explanation than had been previously offered.

I know, forsooth, that some objections may be made to percussion applied to the diseases of the heart. The volume of this organ varying with individuals, there must be an uncertainty as to whether the extent of the space in which the flatness is found, be normal or otherwise. But what physician will not be able to distinguish whether this extent surpasses the reasonable and probable volume of the heart, in the patient whom he examines? And besides, the variations in the volume of the heart never reach the limits which would be considered pathological by the practitioner, who does not think of percussing the chest until the general symptoms have already fixed his attention. In the second place, will percussion permit us to distinguish an enlargement of the heart from an accumulation of serum in the pericardium? Certainly not, if we have reference to its results alone. But explore with the hand the pulsations of the heart, study its rhythm and compare it with that of the pulse; consult the general and sympathetic symptoms which the patient presents, and you will approach some degrees nearer the truth. What then is the method of exploration which alone can replace all others, and which does not require to be combined with them, to afford more certain results? Auscultation itself, the one whose results have been the most brilliant, cannot do without this union with the others; how much less, then, should percussion!

Again, Corvisart is far from confining himself exclusively to percussion, and he studies the other symptoms with a care unknown before his time. Read what he says of the *facies propria* of patients affected with a lesion of the heart; of the engorgement of the venous system throughout the whole body; of the jugular pulse, which he wishes to be well distinguished from that of the subjacent carotids; of the pulsations of the heart, often perceptible to the sight, and accompanied by a *sort of roaring* (*bruissement*) *or peculiar disorder of the circulation when there is contraction of the orifices* (does this not anticipate the bellows sound (*bruit de soufflet*) of Laennec?); of the prominence of the walls of the chest, which he first pointed out under the name of *arching* (*voussure*) of the thorax; of the engorgement of the liver, which so often accompanies these lesions; of the swelling of these limbs, caused by the blood not being able to return to the abdomen (Corvisart, op. cit., p. 373 et seq.); and say whether he has neglected any of the elements suitable for conducting him to the object of his researches—to recognise during life the diseases of the heart. He has not indeed removed all difficulties, he has not made all uncertainties disappear; these difficulties and uncertainties still exist, and we shall shortly inquire whether auscultation has produced any very satisfactory results, for the solution of all these questions.

From all that I have just said, I believe myself right in concluding, that before the time of Laennec the theory of the diseases of the heart had been pushed farther, and was more advanced than that of certain diseases of the lungs and pleura, and that it was also easier to recognise an aneurism of the heart, than certain chronic pleurisies and consumption. What was not to be expected, then, as the complement to the art of diagnosis applied to those diseases, from the brilliant discovery of Laennec! Alas! these flattering hopes have been disappointed: and if the pathological anatomy of the heart has made recent improvement, the diagnosis of those diseases has remained at or near the point where Corvisart left it.

Whence this immense difference between the results of auscultation applied to diseases of the heart on the one hand, and to those of the lungs on the other? Why so much uncertainty in the former, and so much certainty in the latter? All arises from the difference in the starting point (*point de départ*). Auscultation of the lungs, indeed, was founded on this simple and exclusive basis, that every modification of the normal sound of respiration, denoted some obstacle to the respiratory act being executed as in the healthy state. This admitted, it was only necessary to find anatomically to what lesion such a sound or râle, brought to the ear of the observer by the stethoscope, corresponded. This has been effected; and if the new symptomatology which has resulted from it, has been found to be immovably certain, it is because the principle on which it was founded is itself beyond controversy. The respiratory murmur could only be attributed to the dilatation of the pulmonary vesicles by atmospheric air: so evident is this, since it results from the very functions of the lungs, that no other explanation could be given. This respiratory murmur, always the same in the normal state, could only be modified in an uniform manner by the same lesion in whatever individual it should be observed, and this modification of the sound could only be in constant relation with the nature of the obstacle which this lesion induced in the respiratory function. Hence, as I have said, the certainty of the new signs which Laennec has appointed to diseases of the chest.

But for the diseases of the heart, there is unfortunately nothing similar; and, in the first place, there is no fixed starting point to enable us to interpret in a certain manner the modifications which the sounds of the heart may present. In short, to assign a certain and real value to these modifications, it would be necessary to agree on the cause of the sounds, and on this point there are almost as many different opinions as there are authors.

Laennec has no where given his views respecting the cause to which he attributed these sounds; but it is easy to see, from the complacency with which he analyses the experiments of Erman and Wollaston on the sounds produced by the contraction of certain muscles, that it is to the contraction itself of the ventricles and auricles, that he attributes the double sound which accompanies the beatings of the heart. According to him, the first and duller sound, synchronous with the pulse, is produced by the contraction of the ventricles; the second, sharper (*éclatant*), and immediately following the first, results from the contraction of the auricles; and the short, very well marked interval of time, which separates this double sound from that which follows, corresponds with the repose of the heart. (Laennec, op. cit., vol. ii, p. 404, et seq.)

This theory, which for a long time met with no opposition, as if the great name of its author had served as a bulwark against all criticism, was slightly modified by M. Marc Despine who, in a memoir read before the Academy of Medicine, the 29th July, 1831, arrived by exclusion at this result: that the first sound was owing to the contraction of the ventricles, and the second to their dilatation. But after all, the original idea of Laennec's theory still existed, and the contraction of the fleshy fibres of the heart was recognised as the cause of the sounds of this organ. All at once this theory found opponents; and so soon as the signal was given, new theories were started in rivalry, agreeing among themselves only in the complete subversion of the ideas of Laennec.

M. Pigeaux was the first who commenced this kind of scientific insurrection against the illustrious author of auscultation, in a thesis defended at Paris in 1832, entitled: *Diverses propositions relatives à la physiologie et à la pathologie du systéme circulatoire;* and in a memoir entitled: *Examen critique et comparatif des divers systèmes qui ont été émis sur la cause des bruits du cœur.* (*Archives générales de Médicine*, vol. xxx, p. 356, November, 1832). The object of M. Pigeaux in these different works was to show that the first sound, which he calls the *inferior*, was produced by the shock of the blood thrust by the auricle against the walls of the ventricles; and that the second or *superior sound*, was produced by the shock of the same liquid, against the walls of the aorta and of the pulmonary artery. But in announcing this opinion, M. Pigeaux did not reflect on the flagrant contradiction which it gave to facts. For, if the first sound be due to the shock of the blood against the walls of the ventricles, and correspond consequently with the contraction of the auricles, how happens it that it is synchronous with the pulse? As the arterial pulsation must evidently coincide with the sound resulting from the contraction of the ventricle, it should be synchronous with the second or *superior sound.* Now, that the contrary takes place cannot be denied, and this single fact annihilates the theory of M. Pigeaux. Hence, in p. 49 of the work recently published by him under the title of *Traité pratique des maladies du cœur* (Paris, 1839), this author has modified his theory so as to make the first or *inferior* sound arise from the friction (*frottement*) of the blood against the parietes of the ventricles, the orifices and walls of the great vessels; and the second or *superior* sound, from the friction of the blood against the walls of the auricles, the auriculo-ventricular valves, and the cavity of the ventricles. This new theory, which completely removes the objection that I have just named, has besides the merit of agreeing pretty well with the mechanism of the contractions of the heart, such as it is admitted by all physiologists. But this is not sufficient to convert it from a mere scientific opinion to an incontrovertible truth, and I shall not consider the arguments by which the author pretends to arrive at that result.

Dr. Spittal, whose experiments relative to the crepitant râle of pneumonia I have already mentioned, has put forth an idea nearly similar to that of M. Pigeaux, on the cause of the sounds of the heart, which he likewise explains by the friction of the blood against the cardiac walls. According to this author, the movement of the fluids on the solids determines the production of the sound; and he founds his opinion on an experiment in which, placing the ear on a small leaden tube, which conducted water to a faucet, every time that the latter was opened he heard a very distinct rubbing sound, whose intensity was always in proportion to the degree of the opening of the faucet; and by opening and closing it alternately, he obtained a sound altogether similar to the rasping sound sometimes heard in certain diseases of the heart.

Dr. Hope, author of an excellent treatise on diseases of the heart, published in 1832, has adopted an opinion on the cause of the sounds of the heart, which differs from the preceding, inasmuch as it refers these sounds to the collision of the columns of blood in the interior of this organ. At the moment of its systole, the layer of liquid in contact with the ventricles receives an impulsion which, by propagation from particle to particle, produces the first sound. This is reinforced by the collision of the numerous currents which escape from the interval of the columnæ carneæ of the heart. At the moment of the diastole, the blood coming from the auricles rushes with violence into the ventricles; but the dilatation of the latter being suddenly stopped, this determines a reaction against the liquid admitted into the ventricular cavity: hence the production of the second sound.

About the time of the publication of the writings that I have just named, M. Rouannet maintained, in a thesis entitled: *Analyse des bruits du cœur* (Paris, 1832), that the sounds of the heart are owing to the play of the valves of that organ, the first sound being produced by the auriculo-ventricular valves, during the contraction of the ventricles, and the second taking place at the moment when the elastic reaction of the aorta and pulmonary artery replaces the sigmoid valves of these vessels. This theory, the idea of which M. Rouannet derived from Carswell, and which was adopted by M. Bouillaud in his *Traité clinique des maladies du cœur* (1835), and by M. Ph. Bérard, in his article *Cœur* (*physiologie*) in the 2d edition of the *Dictionnaire de Médecine* or *Répertoire général des Sciences Médicales*, is supported by reasons and experiments which render it infinitely more probable than the others. However, I should remark, that it is open to a serious objection, of which its adversaries have taken great advantage: it is that, though explaining pretty well the normal sounds of the heart, it is insufficient for the pathological sounds, which cannot, in scarcely any case, be referred to the play of the valves. M. Rouannet and his partisans are therefore forced, in order to make these abnormal sounds agree with their theory, to admit another element in the production of the sounds of the heart, viz. the friction of the blood against the walls of the cavities containing it. This objection is more specious than solid, but it is not my business to refute it; I will only draw from it a new proof that there is no evident and indisputable connection between the sounds of the heart and the cause which produces them, since we have already stated on this point very many opposite opinions, not one of which presents those convincing characters which unite all suffrages.

But we are not at the end of all these theories, and I continue the review. M. Magendie read before the *Académie des Sciences*, at its sitting of 3d February, 1834, a memoir, in which he endeavors to prove that the double sound of the heart is produced by the successive collision of the apex and the base of this organ against the anterior wall of the thorax. The theory of M. Magendie was destroyed as soon as published, by M. Bouillaud, who having laid bare the heart of a cock and that of a rabbit, and having completely separated the organ from the solid parietes of the chest, distinctly heard the double sound. (Bouillaud, op. cit., vol. i, p. 123 et seq.)

Finally, M. Piorry has inserted in the *Archives Générales de Médicine* (June 1834) an article in which, refuting by contrary experiments those on which M. Rouannet has founded his theory, he arrives at this result: 1. that the play of the valves does not produce the sounds of the heart; 2. that the sounds of the right heart are louder than those of the left; 3. that he is tempted to attribute the dull sound to the contractions of the left heart, and the clear sound to those of the right. In writing this, M. Piorry forgot that the contractions of the ventricles are simultaneous, and that the two sounds are not.

My object in thus enumerating at length the different theories which have been promulgated on the cause of the sounds of the heart, has not been to discuss which is the best, but only to show how widely they differ from each other. It was, however, indispensable to agree on the cause of this sound, in order to interpret in an uniform manner the modifications perceptible by the stethoscope. We shall, therefore, find the same discrepancy among authors, on the value of the abnormal sounds of the heart.

All these abnormal sounds consist of the bellows murmur, the rasping or sawing sound, and the purring tremor (*frémissement cataire*). Let us see what has been said of them by the authors who have attempted their explanation.

Laennec thus commences the chapter which he has devoted to them: "The phenomena of which I am about to speak are so much the more remarkable, that among all those which mediate auscultation has made known, *they alone are not connected with any organic lesion to which they can be referred.*" (Op. cit., 2d edition, vol. ii, p. 421). And, in truth, in reading this chapter it is evident that Laennec regarded these different sounds as nervous phenomena only, the results of a particular modification of innervation. Their semeiotic value is therefore nothing, and it is impossible to draw from them any induction for the *localisation* of an affection of the heart.

Dance, who in his article *Auscultation*, of the new *Dictionnaire de Médicine*, has followed Laennec step by step, without formally explaining himself on the various sounds of which we are speaking, acknowledges that the stethoscopic signs in diseases of the heart, are far from having the certainty they possess in those of the lungs, and that they should be regarded so far only as they agree with the general symptoms which can never vary.

M. Bouillaud, whom we have seen adopting the opinion of M. Rouannet on the sounds of the heart, and like this physiologist, referring them to the play of the valves, regards the different abnormal sounds of which we are speaking, and especially the bellows sound, as pathognomonic of contractions of the orifices. The column of blood coming from the auricle, forced to traverse the contracted orifice more rapidly in order to gain the ventricle, roars and murmurs like a river which, after having flowed tranquilly and silently, suddenly meets with an obstacle. (Bouillaud, op. cit., vol. i, p. 173). But as he could not be ignorant that too many authentic facts were opposed to the reference of the bellows sound, in all cases, to an organic contraction of the auriculo-ventricular orifices, he states further on, that he has likewise found the bellows-sound in cases of polypi of the heart; in those of fibrinous concretions formed in the ventricles, at a period evidently anterior to death; in cases of hypertrophy of the ventricles, the orifices not being contracted; and lastly, in a case of hemorrhage which subsequently produced the death of the patient (at the autopsy the heart was found healthy). In all these cases M. Bouillaud explains the bellows sound by an excessive friction (*frottement*) of the blood against the parietes of the orifices. Whatever be the value of this explanation, it nevertheless follows that M. Bouillaud has observed the bellows sound in too great a number of different cases, for it to be pathognomonic of any one in particular.

Finally, M. Pigeaux terminates his observations on these sounds by the following conclusions: "To recapitulate, we see, 1. that all the abnormal sounds, far from being other than the physiological, are but a simple modification of them, resulting from a friction increased either by the unusual rapidity of the blood, or by the asperities, and the rigidity of the valves which oppose its passage; 2. that the normal sounds are simply veiled by the pathological, but they exist notwithstanding; 3. that the abnormal sounds have no absolute value for denoting this or that alteration of the valves, and that they do not exist even when the valves are altered to the greatest extent."

In view of these facts, am I not authorised to say with M. Littré, author of the article *Cœur* (*Pathologie générale*) in the 2d edition of the *Dictionnaire de Médicine:* "As for the permanent rasping and bellows sounds, they denote in general an alteration of the orifices, either arterial or auriculo-ventricular. But what alteration, and of what orifices? There lies the difficulty. *It is clear that the explanations which will be given, will depend on the theory which may be adopted of the sounds of the heart.*"

The reflection of M. Littré is so just that, although published in 1834, it is still confirmed by the diversity in the new theories which are incessantly brought forward for the explanation of these various sounds. Thus M. Beau has inserted in the *Archives générales de Médicine*, for 1838, an article entitled: *Recherches sur la cause des bruits anormaux des arteres, et application de ces recherches à l'étude de plusieurs maladies, et principalement de la chlorose*, in which, adopting the opinion already promulged by several physiologists, that the abnormal sounds of the arteries are owing to the thrill (*frémissement*) produced by the wave of blood on the arterial parietes by an exaggerated friction, he establishes as a principle, that the indispensable condition for the production of vibration in the tubes is the presence of a mass of liquid too great for the capacity of the vessel. On this principle he endeavors to prove that in all the diseases in which the abnormal arterial sounds have been observed, this disproportion exists between the calibre of the vessels and the quantity of blood which traverses them. This is easy in simple aneurisms, or in aneurismal varix. But applying his theory to other diseases in which the bellows or other sounds of the arteries have been heard, he draws the conclusion that, in *plethora*, there is a continued superabundance of blood capable of producing this disproportion; in *hypochondriasis* there is likewise a superabundance of blood, but temporary; that *chlorosis*, far from being a variety of anemia, is much rather produced by an exuberance of blood, differing from plethora in this particular, that the blood is deprived of a part of its nutritive qualities, and consequently discolored and more abundant in serum: an opinion, be it said in passing, rather contradictory to facts, and which M. Beau would find very difficult to prove.

Another writer, M. Delaharpe of Lausanne, treating the same subject in a memoir inserted in the *Archives génér. de Med.*, of August and September, 1838, under the title of *Nouvelles recherches sur le bruit de soufflet des artères*, has arrived, by experiments which it is useless to relate here, at this result: that the density of the blood plays a very great part in the production of the bellows sound of the arteries; that this sound is, in general and all things equal, the more intense as the blood is less thick and its current more rapid; that this sound is produced in the artery by the current of liquid which traverses it, precisely in the same manner that the sonorous waves and vibrations arise in a column of air which traverses a tube.

Starting from this principle, the author denies that the bellows sound can be heard in cases of *plethora*, and thinks that there has been some error in the observations in which this sound has been noticed, and that the bellows sound was referable to some other affection. In cases of hypocondriasis, where the bellows sound has also been observed, it is owing to the diminution of the density of the blood, and to a great augmentation in the rapidity of the circulation. *Anemia* and *chlorosis* afford the most favorable circumstances for the production of the abnormal sounds of the arteries, by the thickening of the blood and the diminution of its quantity.

Thus then, two physicians investigating the same abnormal sound observed in the same diseases, but each explaining it after his own manner, have arrived at diametrically opposite results: M. Beau, that chlorosis is produced by an exuberance of blood which at the same time is much more serous than in the natural state; and M. Delaharpe, that in chlorosis there is thickening of the blood and diminution of its quantity. Which is to be credited? and who shall solve the problem?

Truly, he would have great trouble and extreme difficulty who should attempt to disentangle all these opinions for the purpose of deducing from them a certain theory, which universal reason might sanction, and which we might hope to see confirmed by experience. But I have not yet told all; and as if it were not enough that it is morally impossible, in the actual state of the science, to know to what organic lesion of the heart or of the vessels we should refer the abnormal sounds which occupy us, Dr. Graves, in a clinical review inserted in *The Dublin Journal of Medical and Chemical Sciences* (September 1834), cites a case of pneumonia which he observed with Dr. Marsh, and which presented an extremely loud bellows sound, which was heard over all the anterior portion of the chest, but which could not be perceived in the carotids. This bellows sound followed exactly the progress of the disease, offered a greater intensity in proportion as hepatization advanced, and diminished as resolution was effected. The patient was very stout, had always enjoyed perfect health, and had none of the characters of a nervous temperament. Thus, if the observation of Drs. Graves and Marsh should be confirmed, the bellows sound will no longer belong specially to diseases of the heart. This is a new cause of uncertainty for him who seeks to found his diagnosis on the results furnished by auscultation; and still, I have not mentioned here the encephalic bellows sound observed by Dr. Fisher, of which I shall hereafter speak, for it is very evident that this could never be a source of error in diagnosis, at least in diseases of the heart, this sound evidently having its seat in the arteries of the brain, and never to be referred to the central organ of the circulation.

Thus far I have spoken of the bellows sound only; but by the unanimous consent of authors, the sawing, rasping and cooing (*roucoulement*) sounds are only modifications of the first, although they can not be constantly attributed to the same cause. The practitioner who has most distinguished himself by his laudable efforts to arrive at an interpretation of the organic affections of the heart by physical signs perceptible by means of the stethescope, and capable of aiding the diagnosis, M. Bouillaud, in striving to determine the modifications which produce this or that variety of the bellows sound, makes this remarkable avowal: "I greatly fear that the time has not yet arrived for the complete and definite solution of so delicate a question." (Op. cit., vol. i. page 166). He nevertheless proceeds to the explanation of these sounds, and says that the rasping, filing or sawing sounds seem to co-exist with an osseous or cretaceous induration, rather than with a fibrous or fibro-cartilaginous induration, coinciding with considerable diminution of the orifices and with strong and tumultuous movements of the heart. But this opinion, although supported by the great authority of M. Bouillaud, one of the authors who have most contributed in France to the advancement of the pathology of the heart, is nevertheless merely personal, and, although probable, can not be received as a demonstrated truth.

Still, if the number and the nature of these various abnormal sounds were well determined, and it were with them as with the pulmonary rhonchi, which, since Laennec described them, have always been found the same by all practitioners, perhaps we might hope soon to assign them an invariable semeiotic value. But such is not the case, and from time to time we see some observer point out some new sound not yet described, and of which no one again speaks afterwards. Such are: 1. the *musical sibilant râle* which Laennec occasionally observed, and which we find in musical characters in pages 424 and 426 of his vol. ii, (2d edit.), and of which I do not know that any one has since spoken; 2. the sound compared to the *hissing of a serpent* by Dr. Fischer, who observed it in a patient who thought he had some living thing in his breast; this sound was sufficiently loud to be heard at a distance; at the autopsy nothing remarkable was found, save a solid polypous concretion which filled the right ventricle, the auricle of the same side, and the vena cava, (*Journal de Hufeland*, 1821, vol. lii. page 3); 3. the sound analogous to the *croaking of a frog*, observed by Dr. Maas in a patient who was found on examination to have hypertrophy of the ventricle, with attenuation of the parietes of the right cavities and organic alteration of the walls of the aorta (*Journal de Hufeland*, 1826, vol. lxii. page 123); 4. and lastly, the barking sound (*bruit de hurlement*) described by Dr. Puchelt in 1833 (*Annales Cliniques de Heidelberg*, vol. ix. number 4). M. Puchelt had observed it in two patients who, at the autopsy, were found to have hypertrophy of the heart, accompanied with an aneurismal dilatation of the aorta.

This author regards his barking sound as the same which has been pointed out by Laennec, under the name of the *musical sibilant râle;* by M. Fischer, under the name of *hissing of a serpent;* and by M. Maas, under that of *croaking of a*

*frog.* But these different sounds have been compared by the several observers to things too opposite for us to believe that they are the same, and I do not see how Laennec could have musically written the hissing of a serpent or the croaking of a frog. If, however, these sounds are evidently not the same, may they at least be referred to the same lesion? M. Puchelt does not hesitate to do so, and he places the cause in the aneurismal dilatation of the aorta at its origin, and in the organic alteration of its parietes. But in publishing this opinion, M. Puchelt did not reflect, that in the results of the autopsies mentioned by M M. Fischer and Maas nothing similar is noted.

But let us suppose that demonstrated which we have just proved not to be so: let us suppose that each of these abnormal sounds has a determinate and invariable value; how shall we distinguish in which side of the heart it is produced, and consequently which is the diseased side? Laennec has laid it down as an almost invariable rule, that the movements of the left cavities of the heart are principally felt between the cartilages of the fourth and seventh left sternal ribs, and those of the right cavities under the inferior part of the sternum, "so that in case of disease of one side of the heart, the analysis of the pulsations of this viscus, affords results altogether different in the two points." (Op. cit., vol. ii. p. 386). Most authors who have written on this subject, have adopted the opinion of Laennec; and it is morever on this distinction between the points where the beatings of the two ventricles are heard, that M. Bouillaud founds in part his differential diagnosis between hypertrophy of the right ventricle and that of the left. (Bouillaud, vol. ii. p. 447.) But another author whose name is of no little weight in auscultation, Dr. Spittal, is of a contrary opinion, and thinks that, in most cases, we cannot arrive at a precise diagnosis; and that those in which the affected side has been accurately determined are only rare exceptions.

In an article entitled: "*Remarks on the following question: Is it possible, in a case where there exists a dilatation or a hypertrophy of one side only of the heart, to discover by auscul-*

*tation which is the diseased side?*" and inserted in the *Edinb. Med. and Surg. Jour.* (January, 1834), this physician boldly decides in the negative, and in support of this opinion refers to a case in which all the symptoms denoted that the left side of the heart was diseased, and yet, at the autopsy, this side was found quite healthy; but as a compensation, the right side presented a considerable dilatation and hypertrophy. And as a single fact was not sufficient to prove this opinion, he has had the patience to collect eighty cases of diseases of the heart, from the works of Laennec, Bertin, Bouillaud and Dr. Hope; and has found the indication of the diseased side in eighteen cases only, among which but four furnished on dissection a confirmation of the diagnosis.

The conclusion of Dr. Spittal is severe; I must even say that it appears to me exaggerated. I think that an excellent means of ascertaining the diseased side of the heart, is that which M. Littré has pointed out in an article inserted in the *Gazette Médicale de Paris* (1834, p. 499). It consists in listening to the sounds of the heart, not only at the precordial region, but also over the whole chest. We shall then find that if on one side we hear the abnormal sound, on the other we most frequently hear the regular double sound without alteration. The diseased side is therefore denoted by the side of the chest where the abnormal sound is heard. If this sound be heard alike on the right and left, we must conclude that both sides of the heart are equally altered. There would probably not be an exception to this rule save in the very rare cases in which the abnormal sounds of the heart are so loud, that there is no need of the stethescope to hear them. The precepts of M. Littré are very wise, founded on observation; but still this is only an element of progress, and not a real improvement to be noticed in the pathological history of the heart.

Lastly, auscultation does not furnish more certain and more invariable data for the diagnosis of diseases of the aorta. Thus Dr. Hope had laid down a rule, for a long time admitted without exception, that, when the bellows sound is louder over the course of the descending aorta than opposite the valves, and when at the same time it is superficial and accompanied by a hissing sound, it is always the result of disease of this vessel; but Dr. Henderson has reported in the *The Edinburgh Medical and Surgical Journal* (January, 1835) two observations in which a bellows sound, offering all the characters that we have described, coincided with a dilatation of all the cavities of the heart, the parenchyma of which was at the same time hypertrophied: *the aorta was perfectly healthy.* Continuing his researches on this subject, the same author, in an article inserted in the same Journal in 1835, proves that the sounds furnished by aneurisms of the arch of the aorta, vary according to the form, the presence or absence of coagula in the interior of the aneurisms, and according to the extent of the opening by which they communicate with the aorta: aneurisms obstructed and filled with coagula, furnish by themselves no sound, and transmit but one which comes from the heart. When the aneurism produces one which is peculiar to it, it is a rasping sound, that is to say, in other terms, that these sounds yielded by aneurisms of the aorta are susceptible of being modified by so many causes wholly inappreciable to the observer, that it is impossible to rely solely upon them in establishing a diagnosis of the diseases which we are studying.

I might, perhaps, here take leave of this discussion respecting the uncertainty of the stethescopic signs of diseases of the heart, thinking its source had been sufficiently demonstrated; but I should not think my task finished, if I did not succinctly touch upon the principal diseases of which the heart and its appendages may be the seat, to find what their diagnosis has gained by the discovery of Laennec.

### *Pericarditis, acute and chronic. Hydro-Pericardium.*

According to all authors pericarditis has ever been one of the most difficult diseases to recognise. "I have sometimes seen pericarditis *guessed*", says Laennec, "and I have occasionally *guessed* it myself; for I do not think we can employ the word *recognise,* when there are no certain signs, and it happens that we are as often deceived as we guess aright." (Op. cit., vol. ii, page 660.) Corvisart, who likewise admits the extreme difficulty of the diagnosis of this disease, gives as a reason for it, that it runs its course too rapidly to allow the determination of its character; that it is almost always complicated with inflammation of the neighboring organs whose symptoms conceal its own, or which at least mingling with them, form a multiplicity of phenomena in the midst of which it is very difficult to know which is the principal disease. (Corvisart, *Essai sur les maladies organiques du coeur*, p. 5). However it may be with this explanation, it is certain that the præcordial pain, which is ordinarily described as lancinating, pungent, and very severe, is sometimes wanting or is so slight that, to excite it, percussion of the thorax, or pressure on the epigastrium must be resorted to; either it is masked by some other rheumatic, pleuritic or articular pain, or the patient is entirely free from suffering. The augmentation in the force of the pulsations of the heart, their irregularity, their intermittence, the dyspnœa, are also signs of pericarditis which are so frequently wanting that Laennec declares: "That a certain degree only of confidence is to be placed in these signs, even when they are all united; for not only may pericarditis exist without them, but the whole may likewise be present without there being pericarditis." (Op. cit., vol. ii, p. 663). Will auscultation remove this difficulty in the diagnosis? Let us again hear Laennec: "I must admit that mediate auscultation does not give much more certain signs of pericarditis, than the study of the general and local symptoms." (Op. cit., vol. ii, p. 662). And indeed, he indicates no stethoscopic sign belonging specially to this disease. M. Collin however in his thesis entitled: "*Des diverses méthodes d'exploration de la poitrine, et de leur application au diagnostic de seș maladies*" (Paris, 1823), had before spoken of the sound like *new leather* (*bruit de cuir neuf*) as properly belonging to acute pericarditis. This sound has since been observed by M. Bouillaud, who compares it to the grating sound (*bruit de frottement*) which often accompanies the first stage of pleurisy, and which Laennec himself has indicated under the name of *murmur ascensionis et descensionis.* Several English observers have pointed it out, among others Drs. Hope, Stokes and Watson. The latter, in an article published in the Lancet of 1836, has asserted, that whenever during the continuance of rheumatism a grating sound was heard in the precordial region, we might be sure that an acute pericarditis was being developed; that if this sound continued until the death of the patient, the pericardium would be found covered with a rough, non-adherent layer of lymph, at least in the greater part of its extent; and that if it ceased, and did not reappear, it was owing to the pericardium having contracted adhesions in the greater part of its surface by which the friction was prevented. The opinion of Dr. Watson, a little exaggerated perhaps, on the constant formation of adhesions between the laminæ of the pericardium when an effusion has taken place there, has been since contradicted by Dr. Root, who thinks that, when the grating sound has revealed an effusion in the pericardium, it is not impossible that the latter should return to nearly its normal state, save however the formation of those white blotches of variable size, so often observed on the heart, and which he regards as the signs of an inflammation of the pericardium terminated by a partial though not entire absorption. It results therefore from the observation of these different authors, that the grating sound denotes in general, acute pericarditis, with a slight effusion; but unfortunately it does not constantly exist.

It would appear, from these writers, that the same sound is heard in the case of those milky patches of the heart which so often follow pericarditis. But the adhesion of the pericardium to the heart, another pretty common result of this disease, is not disclosed by any symptoms, and appears to be much more innocent in itself, than Corvisart thought. As to its being a frequent cause of dilatation of the ventricles, as asserted by M. Beau in a memoir published in the *Archives*

*de Médicine,* April, 1836, no one certainly will believe it, if he has reflected ever so little that the facility with which considerable effusions are formed in the pericardium, is the best proof of the extensibility of that membrane, a physical property diametrically opposed to that which would be indispensable for the production of the effect indicated by M. Beau.

Chronic pericarditis is not of easier diagnosis than the acute, but its most ordinary effect is an accumulation of pus in the serous envelope of the heart. Now, in this respect, its diagnosis is confounded with that of hydro-pericardium, although, in the eyes of the pathologist, there is a vast difference between an accumulation of pus, and one of limpid serum in a serous cavity, and it would be impossible for him to attribute both to the action of the same cause.

I shall not stop to discuss the quantity of serum necessary for one to say there is hydro-pericardium: it is not my object. I hasten to the pathognomonic signs indicated by authors for this disease; the principal are: the sensation of an enormous weight in the precordial region (Lancisi); the sensation of the heart swimming in a great quantity of water (Reimann and Saxonia); the inability of the patient to lie on the right side, without being on the point of suffocation: all signs which, according to Morgagni, are scarcely worthy of being put on the list even of equivocal signs of this affection. As much might almost be said of those which Corvisart indicates in this passage: "In patients affected with hydro-pericardium, the countenance is habitually violet, the lips black and livid; they experience a painful anxiety, an irksome weight in the precordial region, a difficulty of respiration which threatens suffocation whenever an attempt is made to assume a horizontal posture; syncope frequently occurs; etc." (Op. cit., p. 41). Sénac has seen in the intervals of the third, fourth, and fifth ribs, the undulations of the effused liquid. (Sénac, op. cit., vol. ii, p. 364). Corvisart says, he has recognised them by the touch, but only in one case, and yet he dares not affirm that he really felt the wave of the effused liquid, and not an undulation produced by the pulsations of the heart: "But I

feel assured," he adds, "that if it be thus, the particular character of these pulsations is very perceptible." (Op. cit., p. 42). M. Bouillaud says, with reference to this passage, that he has once had occasion to make an analogous remark. He thought, at first, he had ascertained the existence of fluctuation in the precordial region; but an attentive examination soon convinced him that the phenomenon taken for fluctuation was nothing else than the contraction of the heart, which had been pushed from its accustomed place, and applied as it were against the thoracic parietes by an enormous tumor situated in the left side of the chest. (Bouillaud, op. cit., vol. ii. p. 336).

But Corvisart has drawn from percussion of the thorax (a method which he brought to such perfection, that more honor has accrued to him, than even to Avenbrugger its inventor), signs much more positive, than those just enumerated: "When percussion of the chest is practised," he says, "whether the patient be sitting or placed horizontally in his bed, the sound yielded by this cavity is obscure, and indeed absent anteriorly and on the left, over an extent proportioned to the dilatation which the liquid causes in the pericardium, In some cases the left side of the chest is more elevated, more rounded and more prominent than the right, etc." (op, cit., p. 4). Dulness of the precordial region and prominence (*voussure*) of the same, are the new signs first indicated by Corvisart, and they have since been noticed by all observers. They are really of great value, but are yet insufficient to show incontestably that there is hydro-pericardium. Who shall prove, indeed, that this flatness of the precordial region is not owing to an augmentation of the volume of the heart? . . . . . Corvisart afterwards adds, that a sign which appears to him to merit more confidence than all others, is the sensation of the pulsations of the heart in different points of a pretty extensive circle, which would not take place if the heart was retained by the pericardium as in its ordinary state. (Op. cit., p. 43). Here Corvisart no longer agrees with other observers, who appear unanimous in saying that in cases of hydro-pericardium, the hand applied on the precordial region does not feel the pulsations of the heart.

Will auscultation finally decide this question? Will it solve the problem of the diagnosis of hydro-pericardium? Let us hear Laennec: "In these cases the stethescope will doubtless aid in establishing a diagnosis, but I cannot say what signs it will furnish, because I have not had sufficient opportunities of observing idiopathic hydro-pericardium." (Op. cit., vol. ii, p. 670). Bouillaud says that when the fluid in hydro-pericardium is abundant, "the sounds of the heart are distant, obscure and resemble somewhat those of the fœtal heart." And in support of his opinion he cites a passage from a case of hydro-pericardium communicated to him by M. Casimir Broussais, in which, "only feeble and obscure contractions were heard, and the more obscure as the distance was greater from the region corresponding to the base of the heart; so that in this point the two contractions were distinct, whilst at the extremities of the region occupied by the flat sound a sort of murmur only was perceived, somewhat analogous to that heard when a shell is held to the ear." (Bouillaud, op. cit., vol. ii, p. 338).

Theory long before indicated this obscurity in the sounds of the heart, which to reach the ear traverse the fluid poured out into the pericardium; but if we reflect how the intensity of these sounds varies in different persons, according to the greater or less embonpoint or other similar cause, we shall be easily convinced how transient this sign is, and consequently of the little value it possesses as it respects diagnosis.

### *Carditis. Endo-Carditis.*

Carditis, or general inflammation of the muscular tissue of the heart, has most often been confounded with pericarditis or with inflammation of the internal serous lining of the organ, which we shall speak of under the name of endo-carditis. This affection, if it really exists separately, and confined to the fleshy tissue of the heart, has as yet manifested itself only by the formation of abscesses in this tissue, which have never been revealed except by dissection. No attempt has yet been made to facilitate their diagnosis by auscultation: I have nothing more to say of them.

Not so with endo-carditis. This affection, of which Corvisart says not a word, and which was regarded as very rare by Laennec, has been carefully studied by M. Bouillaud, who sees in it the cause of all the fibrous, osseous or even calcareous degenerations, which may be presented by the valves of the heart or of the great vessels. I shall not discuss this opinion, connected as it is with the doctrines of the physiological school, which, as is well known, is disposed to consider inflammation the sole cause of every possible organic alteration. The fallacy of this doctrine was long ago shown; a proper estimate has long since been placed on these erroneous and exclusive opinions, which go to strike the changes and decomposition of the fluids from among the causes of abnormal productions in our economy, to refer every thing to an unnatural afflux of blood, whilst this afflux often does not take place, or at least is only momentary, and sometimes is only the consequence of an already advanced stage of organic alteration, instead of being its cause.

Since M. Bouillaud, several authors have treated of endocarditis, among others M. Littré, in the second edition of the *Dictionnaire de Médecine;* Dr. Cazaneuve, of the military hospital of Val-de-Grâce, who has made it the subject of his inaugural thesis, inserted in the *Gazette Médicale* of 1836, (p. 401); and Dr. Watson, in an article on rheumatic carditis, published in the *Lancet* in 1837, etc. The history of this disease being somewhat new, auscultation has been put under contribution like the other modes of investigation, to establish a diagnosis; but, as in other diseases of the heart, it affords only hypothetical data. The signs indicated by M. Bouillaud are: prominence (*voussure*) of the præcordial region, where there is pericarditis at the same time; vibration of this region from the violence of the pulsations of the heart, which assume the character of palpitations; the sensation of a vibratory thrill (*frémissment*), more or less marked in the same region; smallness and slight developement of the pulse, coinciding with the violent contractions of the ventricle; anxiety, jactitation, fainting and syncope.

The region of the heart, in its entire extent, gives a flat sound on percussion. This flatness, coinciding with the violent pulsations of the heart, is thereby distinguished from that which accompanies hydro-pericardium, in which these pulsations are not felt.

After this statement of symptoms, which is sufficiently exact, and conformable to theory, what shall we demand of auscultation? some constant and invariable physical sign, doubtless, which would enable us to recognise the disease in all cases where the general symptoms just enumerated should be wholly or in part wanting? Alas! as to any pathognomonic sign, we obtain the bellows sound only, and sometimes, according to M. Bouillaud, a metallic tinkling synchronous with the systole of the ventricle, and which M. Cazaneuve, who in his thesis has closely followed Bouillaud, attributes to the shock of the heart against the parietes of the thorax. In vain does Professor Bouillaud say, that this bellows sound is stronger as the pulsations of the heart are more violent and precipitate; in vain does Dr. Watson affirm that every time a deep blowing sound supervenes in the course of a rheumatism, it constantly denotes an inflammation of the internal membrane of the heart, particularly of that portion which covers the valves; I shall still refuse to consider the bellows sound a pathognomonic sign of endo-carditis.

Let it be remembered, now, that by the unanimous consent of pathologists, the bellows sound nearly always coincides with contraction of the orifices of the heart. Laennec had remarked this, although he added, that the cases in which the bellows-sound was observed without there being any organic alteration of the heart whatever, were too frequent to make the production of the sound depend essentially on this contraction. (Laennec, op. cit., vol. ii, p. 441). M. Andral, in speaking of the bellows-sound, says, it is very certain that, in a great number of cases its existence coincides with that of an obstacle to the free course of the blood through the different orifices of the heart. He even goes so far as to say that, "according to the place where this sound is heard, and the time of the contraction which accompanies it, we may even assign the precise seat of the obstacle." (*Clinique Médicale,* 2d edition, p. 161). But he afterwards makes the same reservation as Laennec for the numerous cases where the bellows sound has existed in healthy hearts, or has been wanting in hearts presenting the alteration in question. We have seen M. Bouillaud profess the same opinion, and compare this sound to the roaring of a river which rushes forward against some obstacle; but if the bellows sound essentially indicates a contraction of the orifices of the heart, what does it indicate in endo-carditis?

What, indeed, are the anatomical characters of endo-carditis? "Redness, and sometimes softening of the membrane, frequently a *considerable thickening*, which exists very evidently only on the valves, where the membrane is, as it were, doubled and strengthened by a fibrous tissue." (Bouillaud, op. cit., vol. ii, p. 174). But is this *considerable thickening* sufficient to hinder the circulation of the blood through the orifices? this will be doubted, if we reflect that, by M. Bouillaud's own confession, the vegetations of the internal membrane of the heart and of the valves, are revealed by no sign, no sound perceptible with the stethoscope, and that they are, according to the forcible expression of this author, *undiagnosticable.* Now, ought they not to hinder the circulation an hundred times more than simple thickening of the endo-cardium, however *considerable* it may be?

But this is not all. M. Littré has nearly demonstrated, in a memoir inserted in the *Gazette Médicale de Paris* (under the title of *Recherches sur l' insuffisance des valvules auriculo-ventirculaires et des valvules sygmoïdes de l' aorte,* that the bellows sound is likewise an essential sign of this insufficiency, or, to explain myself better, of the regurgitation of the blood through the orifices, during the contraction of the ventricles, when the mitral and tricuspid valves are defective; and during their dilatation, when the semilunar valves are diseased. This insufficiency of the valves may arise, either from ulceration, ossification, or from the orifice to which they are attached being so much dilated, that they can no longer close it completely.

Thus, then, the bellows sound is at the same time a pathognomonic sign of contraction of the orifices, and of their dilatation, which necessarily renders the valves insufficient; and the only character which is given to distinguish the former from the latter, is that: 1. in contractions of the auriculo-ventricular orifices, the bellows sound replaces the second sound, or that accompanying the dilatation of the ventricle; 2. in the case of insufficiency of the valves of these same orifices, the bellows sound is heard at the moment of the contraction of the ventricle, and consequently replaces the first sound. But when the insufficiency of the semilunar valves will permit the blood to flow back from the aorta, during the dilatation of the ventricles, the bellows sound will still replace the second sound: how then are we to distinguish this case from that of contraction of the auriculo-ventricular orifice? M. Littré says that, in the case of insufficiency of the semilunar valves, the bellows sound is prolonged into the aorta (Littré, loc. cit.): it is really expected, then, that we should always establish with mathematical precision the limits of the space where the bellows sound is heard! that we should discover constantly whether this sound does not extend beyond the ventricle, or that it is slightly prolonged in the arteries! Is it not enough to say which of the sounds of the heart is replaced by the bellows sound, and to seize just the moment in which it is heard, in a space of time so short as that of the double pulsation?

To recapitulate, an abnormal sound which is found to be the essential sign of three affections, so different as simple endo-carditis, contraction of the cardiac orifices, and insufficiency of the valves, which is itself often an effect of the dilatation of these orifices, is not the pathognomonic sign of either:

I think that I have sufficiently proved it. I may therefore rightfully conclude that auscultation furnishes no reasonable and sufficient basis on which to establish the diagnosis of endocarditis.

### *Hypertrophy. Dilatation of the Heart.*

These two pathological states, altogether distinct, and perfectly capable of existing separately, have always been confounded under the name of aneurisms. Corvisart himself did not perceive the essential differences which distinguish them from each other, and treats only of aneurisms, which he divides into *active* and *passive*, accordingly as they are accompanied by hypertrophy or attenuation of the parietes of the heart.

Having to treat of these diseases with respect to their diagnosis only, I proceed at once to the symptoms of hypertrophy. M. Bouillaud thus briefly analyses them: "Permanent augmentation of the force and extent of the heart's pulsations, and consequent augmentation of the double normal sound which accompanies them; . . . . increased extent of the dulness in the præcordial region, and sometimes a marked projection, a manifest prominence (*voussure*) of this region." (Op. cit., vol. ii; p. 440). These signs, however, are not applicable to all cases of hypertrophy. Thus, the flat sound of the præcordial region is not obtained over a space greater than in the healthy state, except in *excentric* hypertrophy, the sonorousness of this region remaining the same in cases where the hypertrophy is developed at the expense of the cavity of the heart. It is likewise in the first only, that the pulsations of the heart are increased so as to be perceived by the eye, to raise the hand applied on the præcordial region, and sometimes to shake the whole body. I say nothing of the pulse, because it is subject to so many variations that it would be impossible to draw the least sign from it. Neither do I speak of the dyspnœa, of the disposition to hemorrhagies and dropsies, rational signs which do not belong to hypertrophy more than to the other diseases of the heart. I hasten to take up the results obtained by auscultation.

The stethoscopic signs vary according to the nature of the hypertrophy. If it be concentric, the sounds of the heart are dull, obscure, as it were smothered; but if it be excentric, these sounds are louder, more sonorous, clearer, extending over a greater portion of the chest, and often even to the posterior part of this cavity. In this case a metallic tinkling or clacking is frequently heard in the anterior part of the chest, resulting from the shock of the heart against the posterior part of the *sternum*. (Bouillaud, op. cit., vol. ii, p. 442). To these signs M. Chomel adds the bellows sound: "for," says he, "it has been wrongly contended that the bellows sound belongs exclusively to the contraction of the valves; a great number of autopsies has proved to me that hypertrophy without contraction may also produce it." (Art. *Hypertrophie et Dilatation du cœur*, 2d edit. of the Dict. de Méd., or Répert. génér. des Scienc. Médic., vol. vii, p. 295).

Is it possible by means of auscultation to distinguish the diseased from the healthy ventricle? I shall not recur to this question already sufficiently discussed while examining the opinion of Dr. Spittal who, as we have seen, disagrees with Laennec on this point: I will only add that the stethoscopic signs are sufficient for this, in so far only as they accord with the general symptoms of reaction, which are known to differ greatly in the two cases, and to affect chiefly the encephalon in hypertrophy of the left ventricle, and the organs of respiration in that of the right.

As to the dilatation of the cavities of the heart, if it be accompanied, as it most commonly is, by hypertrophy of the parietes, the signs are those which I have just enumerated, save that the dulness on percussion extends over a larger surface. But if the dilatation be complicated with attenuation of the walls of the heart (an extremely rare case), it will likewise be recognised by the dulness of the præcordial region, and the softness of the heart's pulsations, which are obtuse and as if muffled, and lastly by the general signs which indicate a general languor of the circulation. By means of the diagnosis which I have just indicated, hypertrophy and dilatation of the heart may in general be recognised pretty well; but it must be confessed that, among all these symptoms, those furnished by auscultation are far from having that marked preponderance which characterises them in affections of the lungs. In these the stethoscopic signs are every thing, the general symptoms are but secondary. In diseases of the heart, on the contrary, the stethoscopic signs are valuable so far only as they perfectly agree with the general symptoms. But, in the latter case, this unanimous agreement of all the general and local symptoms does not always prevent error. What more common, indeed, than to observe purely nervous palpitations, perfectly simulating hypertrophy and dilatation of the heart, in patients who have this organ entirely sound! Will the application of the stethoscope, however, serve to distinguish this purely nervous state from the superexcitation of the heart produced by hypertrophy? Not at all! By auscultation we shall in most cases hear that unfortunate bellows sound, the constant attendant of every derangement of the circulatory function, even the slightest, and which is destined to be a stumbling-block to all who shall try to *localise* the diseases of the heart by means of the stethoscope. Many nervous or asthenic affections, as hypochondriasis, nostalgia, chlorosis and scurvy, are known to be frequently accompanied by palpitations which simulate to a certain degree the organic affections of the heart, and which are always attended by the bellows sound. I have already had occasion to say to what diametrically opposite conclusions MM. Beau and Delaharpe had arrived, in the explanation of these facts, by starting from contradictory opinions on the first cause of the bellows sound: I shall not revert to them.

There are certainly means of distinguishing in time palpitations purely nervous from those connected with an organic affection. Thus, the former have but a temporary duration; their course is intermittent; they may finally cease; they do not cause dropsy; the sonorousness of the cardiac region is not modified. But no differential symptom is furnished by auscultation; this is a fact which I wished particularly to establish: and in these diseases, as in the preceding ones, it seems evident to me, that this method of investigation has not added much to what the science already possessed. It is then with reason that M. Chomel has penned these lines, truly remarkable as coming from him: "What has already been said of the phenomena peculiar to hypertrophy and dilatation of the heart, will in general serve to distinguish these two affections from each other. It is, however, necessary to be aware, that at all periods of these diseases, and especially in their advanced stage, it is often very difficult and even impossible to distinguish them; and that, in many cases, the physician who does not wish to hazard his judgment, should limit himself to the announcement of a disease of the heart, *without determining either its special seat or even its nature.*" (Chomel, loc. cit., 2d edition of the *Dict. de Med.*, or *Repert. génér. des Scienc. Medic.*, vol. viii, p. 299).

To be faithful to the terms of the programme, I should now take up the signs furnished by auscultation for the diagnosis of diseases of the great vessels, and particularly of aneurisms of the arch of the aorta. But Laennec himself despaired of arriving at any satisfactory result on this point. "Among all the severe lesions of the thoracic organs," says he, "three only remain without a constant pathognomonic sign to the physician expert in auscultation and percussion, to wit: aneurism of the aorta, pericarditis, and concretions of blood in the heart previous to death." (Op. cit., vol. ii., p. 737). We are indebted to M. Bouillaud for the indication of clear, simple or double pulsations, according to the relations of the aneurismal tumor, and distinct from those of the heart, which are perceived by the ear in a circumscribed space behind the sternum or the cartilages of the right false ribs. (Bouillaud, *Diagnostic des anévrisms de l'aorte*, theses de Paris, No. 146, 23d August, 1823). This sign, which is really valuable, requires to be fortified by the concordance of other symptoms, which it does not enter into my plan to develope here.

I stop here in the inquiry as to what has been effected by auscultation applied to diseases of the heart. I think that

I have amply shown what I said in the commencement of this chapter, that its results, so far as they concern the diagnosis of those diseases, are as doubtful and as little capable of guarding against error, as they are certain and endowed with an immense scientific importance in diseases of the lungs. In the latter, the stethoscopic signs alone reveal the disease, even if it be in a latent state, and have not yet excited any general symptom. In diseases of the heart, they give rise to probabilities by no means sufficient to throw light upon the nature of the malady, and still less upon its precise seat.

Does it follow that we must ever despair of this method applied to the investigation of these diseases? It is not in an age like ours that such a scientific heresy would be uttered. By no means; auscultation of diseases of the heart is not without a future; and we must not despair of seeing it some day effect for them what it has already done for diseases of the lungs. Since Laennec, auscultation of the heart has made undoubted progress; but this progress has chiefly consisted in correcting the errors which escaped his sagacity and penetration. Let observers, therefore, continue their labors, and perhaps success will not be long in crowning their efforts! but let them avoid the obstacles which hitherto have baffled those of their predecessors. These obstacles are found in the too exclusive ideas respecting the cause of the normal sounds of the heart, the only source from which an explanation of its abnormal sounds can be drawn. All the theories which I have developed on the cause of the sounds of the heart, are, in fact, too absolute to be in all respects true. Why should not the contraction of the fibres and of the fleshy columns of the heart, the play of the valves, the friction of the columns of blood against each other or against the parietes of the cavities, and even the shock of the heart against the sternum, equally contribute to the production of the double pulsation? This opinion is not peculiar to me; it is entertained by several physicians, among others by Professor Andral, who, in the additions which he has made to Laennec's work, does not seem far from admitting that, "among the causes to which each author has exclusively attributed these sounds, there is none which may not have its part in their production, but that no single one is sufficient to give rise to them."

I think that it is only from inquiries directed in this way, and made after this mixed and truly eclectic theory, that we can hope for favorable results, which would give to auscultation, in the pathology of the heart, the rank which it occupies in that of the lungs. Time alone will decide whether my hopes are well founded.*

### CHAPTER V.—OF THE NERVOUS DISEASES OF THE CHEST: ASTHMA; ANGINA PECTORIS.

It is not without design that I have reserved these two diseases to be treated of last, as what I shall say concerning them will be but the corollary from all that precedes. Indeed it is only after having firmly established the diagnosis of the acute and chronic diseases of the lungs and heart, that these organs should be interrogated again, to discover by the aid of the stethoscope some organic alteration which might satisfactorily explain those attacks of violent dyspnœa or of præcor-

---

* It is very evident from the whole tenor of this chapter, that the author was unacquainted with the more recent inquiries into the action and sounds of the heart, and with the improvements consequently effected in its pathology. In no other way can we account for the low estimate he places on the value of auscultation applied to the diseases of that organ.

Among these researches, those of Dr. Hope stand pre-eminent. His opinion respecting the sounds of the heart, published in 1832, which is quoted by Dr. Peyraud on page 92, was greatly modified by farther experiments made in 1834–5; these experiments were repeated and confirmed by the London committees of the British Association for 1838–9 and 1839–40, and by Drs. Pennock and Moore of Philadelphia, in 1839: so that now the causes of the cardiac sounds are pretty well understood. It was our design to notice this matter at some length, but want of space forbids. We would refer the reader, who desires further information, to the last edition of Dr. Hope's Treatise on the Diseases of the Heart and Great Vessels, which is now republishing in Prof. Dunglison's *Medical Library and Intelligencer*, with notes and details of recent experiments by Dr. Pennock of Philadelphia. He will not fail to perceive that the *future* of Dr. Peyraud has become the *present*, and that the diagnosis of cardiac affections has attained a certainty which falls little short of that previously acquired in those of the chest.—Jun. Ed.

dial pain, whose nature the ancients absolved themselves from inquiring into by accusing the nervous system.

Not but that a great number of authors have made laudable efforts to find in the dead body the causes of these affections, so inexplicable even at the present day: and if we refer to their assertions, we shall see that there is no lesion of the thorax or of the organs it contains, which may not produce attacks of intermittent dyspnœa. Consolidation of the ribs by ossification of the cartilages which unite them to the vertebral column and to the sternum; ossification of the diaphragm; diaphragmatic hernia; rickets and deformity of the thorax; chronic pleurisy, or else adhesions between the two pleuræ; sojourn of certain foreign bodies in the cavity of the respiratory apparatus; developement of polypous productions and of more or less voluminous vegetations in those same cavities; œdema of the glottis; chronic púlmonary catarrh with contraction of the bronchiæ; compression of the bronchiæ by some neighboring tumor; bony or osteo-petrous concretions in the mediastinum, or even in the pulmonary parenchyma; pericarditis; hydro-pericardium; ossification of the valves of the heart, of the coronary arteries, etc. etc.; what is there that has not been cited as the exciting cause of asthma by some author, supporting himself on some fact? As to angina pectoris, the authors who have treated of it appear to attribute it to organic diseases of the heart and of the great vessels, such as ossification of the coronary arteries, hypertrophy of the ventricles, ossification of the valves, osseous degenerations of the walls of the aorta, or the aneurismal dilatation of that vessel; all alterations which we have seen mentioned for the most part, as causes of asthma; so true is it that these alterations are not every thing in the production of the singular phenomena whose assemblage constitutes these two diseases, if, indeed, these lesions are not the mere effect of them, instead of contributing any thing to their formation!

This opinion, that the organic alterations are the effect, and not the cause of asthma, is not new: many practitioners have adopted it, despairing of explaining by those lesions the intermittent character of the diseases attributed to them. It has been sustained by M. Amédeé Lefèvre, among others, in a memoir entitled: *Recherches médicales sur la nature, et le traitement de la maladie connue sous le nom d' asthme*, an essay which was crowned by the Toulouse Medical Society, at its sitting of 7th May, 1835, and inserted in the *Journal hebdomadaire de Médecine.* But, at the same time that he excludes organic alterations of the heart and great vessels as causes producing asthma, he likewise rejects the opinion which regards this disease as a neurosis, and he thinks that asthma is always owing to a spasmodic contraction of the bronchiæ, which may be produced by all the causes which act, either directly or sympathetically, upon the pulmonary mucous membrane. According to him, the cause of asthma always acts on this membrane, and the affection of the muscular fibres of the bronchiæ is consecutive only.

These ideas resemble those of Dr. Godefroy of Mayence, who, in a memoir on *Asthma essentiel considéré comme névrose des bronches*, attributes this malady to a spasmodic contraction of the muscles of Reisseissen, whence results the diminution of the capacity of the bronchial tubes. But the discussion of the greater or less probability of these theories, is altogether foreign to my object, which is simply to find whether auscultation cannot, in certain cases, aid in discovering the organic lesion which is the occasional, if not essential cause of the attacks of intermittent dyspnœa.

There is a disease which had always escaped the attention of practitioners, because its anatomical characters consist in a mere exaggeration of the natural state of the lungs, and its symptoms are confined to a greater or less dyspnœa, varying in intensity according to a multitude of circumstances. This disease, which Laennec first pointed out, as frequently constituting the essential anatomical character of a great many cases of asthma, till then regarded as nervous, is *pulmonary emphysema.* It is known to consist in the distention of the pulmonary vesicles by atmospheric air, which is accumulated there in much greater quantity than in the healthy state

Sometimes the disease stops here, and is only a permanent, excessive, and preternatural distension of the pulmonary vesicles, and then the emphysema is said to be *vesicular;* at other times the distention increasing, the cells burst, and the air, which in the first case was contained in its natural passages, is extravasated in the surrounding cellular tissue: the emphysema then takes the name of *interlobular*. I shall not examine here, whether, as Laennec thought, the pulmonary tissue is sound, apart from this dilatation; or whether there is at the same time hypertrophy of the parietes of the air cells, as M. Louis thinks, in his article *Emphysème des poumons*, (*Dict. de Med.*, 2nd edit.) I pass on to the signs which enable us to distinguish this disease.

The most constant symptom of pulmonary emphysema is a dyspnœa more or less severe but continual, subject, however, to exacerbations which perfectly simulate the attacks of asthma, I should rather say, which constitute asthma itself. Indeed, like the attacks of asthma, these exacerbations supervene without any known cause, and their duration varies with the cause that produces them; very rare at the onset of the disease, they become more frequent and severe the longer the disease continues, and are frequently accompanied by palpitations, which increase the embarrassment of the practitioner, and sometimes would lead him into an erroneous diagnosis, if Laennec had not indicated certain signs for this affection, all drawn from percussion and auscultation.

The side of the chest, the seat of pulmonary emphysema, yields on percussion a very clear sound; and yet auscultation indicates, over most of the same side, only a complete absence of the respiratory murmur, with a slight sibilant râle from time to time, mingled with that mucous râle which Laennec has compared to the clacking of a valve, and which he regards as the index of the displacement of pearly sputa. This absence of the respiratory murmur will not be mistaken for that produced by a pleuritic effusion, because percussion would then yield a flat sound, instead of the very clear sound which I have named. Neither will it be confounded with that which characterises pneumo-thorax, because in this case, the respiratory murmur extends along the vertebral column, the point where the lung retires, compressed by the air effused in the pleura; whilst in emphysema, the respiratory murmur ceases to be heard in all the emphysematous zone, towards the root, as well as the periphery of the lung. But there are cases in which a *dry crepitant rattle with large bubbles* is heard, which M. Louis has indicated under the name of *sub-crepitant râle.* Laennec had regarded it as indicative of *interlobular* emphysema. M. Louis justly considers it as depending on the pulmonary catarrh which always accompanies emphysema. As to the *ascending* and *descending* rubbing or grating sound (*bruit de frottement*), likewise pointed out by Laennec, it very evidently belongs to emphysema, but cannot be its pathognomonic sign; for an analogous sound has been noticed in the commencement of pleurisy and pericarditis, when these phlegmasiæ are yet without effusion. But of what importance is it? the comparative signs of auscultation and percussion, which I have just laid down, perfectly suffice to establish the presence of pulmonary emphysema, and to distinguish it from other affections with which it is liable to be confounded.

Now, let us admit that Laennec has greatly magnified the number of cases in which asthma had no other cause than pulmonary emphysema; who will not pardon him for this exaggeration, so natural to a mind like his, which discovering an anatomical alteration till then unknown, and being enabled to give it so clear and evident a symptomatology, believed itself authorised in thinking that many cases of pulmonary emphysema had passed unperceived, and had been described as cases of essential asthma? Besides, Laennec has not said that all cases of asthma were emphysema; and as a proof of it, he has devoted a chapter of his work to the study of *spasmodic asthma*, the name under which he designates that form which no anatomical lesion can account for, and which he regards as an essential neurosis.

But pulmonary emphysema is not the only affection that can be detected by means of auscultation, and that is capable of producing attacks of asthma.

Laennec has described, under the name of *dry catarrh*, a disease consisting of a chronic inflammation of the bronchiæ, which is accompanied by no expectoration, or at least by very little. This disease is often concomitant with asthma, in cases where it is not its primary cause. This affection, common in old gouty persons, hypochondriacs, and those whose constitution has been injured by excess, presents as its principal symptom a dyspnœa that affords all the characters of that constituting asthma, and is always confounded with it, inasmuch as it returns by attacks which last several days. Towards the end of these attacks, a cough supervenes, and then the oppression diminishes. After several days, the efforts of coughing produce pearly sputa, whose expectoration causes a still more perceptible diminution of the dyspnœa. The stethoscopic sign which Laennec has allotted to it, is: diminution of the respiratory murmur without a bronchial râle, coinciding with natural sonorousness of the chest. When dry catarrh is protracted during a certain time, it ordinarily engenders pulmonary emphysema, an affection which we have seen to be a frequent concomitant of asthma.

M. Quissac, then chief house-surgeon of Hotel-Dieu at Montpelier, first called the attention of practitioners to a disease previously undescribed, in an excellent monograph published in the *Gazette Médicale de Paris* of 1836 (pages 161 and 177). This disease, to which he gives the name of contraction of the lungs, has for its anatomical character the horn-like induration (*racornissement*) of the lungs, whose density increases in direct proportion to the smallness of the volume to which they are reduced. If this examination be pushed farther, we find that the mucous membrane of the bronchiæ exhibits traces of phlogosis, and that the cavity of these conduits is effaced in their ultimate divisions. The symptoms of this affection may be confounded with those of asthma when they are acute, and are then only distinguished by the brief duration of the malady compared with the essentially chronic course of asthma. But when the contraction assumes a chronic course, the rapid and complete wasting which a want of hematosis induces, would make it rather resemble phthisis;and it is, indeed, among this class of diseases that M. Quissac appears willing to place it, from his calling it *phthisis by contraction.* The stethoscopic signs which he gives to it, are: absence of the respiratory murmur, and great sonorousness of the thorax. These are identical with those of pneumo-thorax, unattended by effusion into the pleura; but it must be confessed that M. Quissac has not sufficiently distinguished these two diseases from each other. It is very evident, in the observations which he cites, that the contraction is essential, and that it does not resemble the simple compression of the lung by an effusion of air or pus. But M. Quissac thinks that the pleura, in this case, contains nothing, not even gas: which contradicts all the received ideas of animal physics. However, this newly described disease requires to be studied anew; and the signal once given, observation aided by pathological anatomy, will do the rest.

Drs. Kopp and Hirsch of Kœnigsburg, were the first to describe a disease which has since been generally called, in Germany, *thymic asthma.* This disease, peculiar to infancy, and the cause of which, as its name denotes, consists in hypertrophy of the thymus gland, is characterised by fits of suffocation during which respiration is almost suspended, and which return periodically, especially at the moment of waking, during deglutition, and when the child cries. The first memoir *ex professo*, on this disease, was read by Dr. Kopp, at the assembly of learned German naturalists at Heidelberg, in September, 1829. From the description given by the authors of the symptoms which characterise this disease, we see at once that it has the name only in common with asthma, and that there would be much more danger of confounding it with hooping-cough. Its physical signs are: five or six inspirations, at first hissing, then deeper and more painful, alternating with a scarcely perceptible respiration, whose sound bears some resemblance to that of croup developed to a high degree. The acute cry heard at the commencement of inspiration is the pathognomonic sign of this affection. Another

German physician, Dr. Roesch of Schwenning (Wurtemberg), who has been much engaged in observing thymic asthma, without denying that hypertrophy of the thymus has been encountered in all cases, does not, however, make it the essential character of this disease; in his view, this essential character consists in a spasm of the glottis. He considers it, therefore, as constituting a true neurosis, and he would be tempted to call it convulsive asthma of the larynx. He founds his opinion on the fact that the symptoms which Drs. Kopp and Hirsch have assigned to it, are not those which would indicate compression of the lung, and a mechanical obstacle to the circulation of the blood and the movements of the heart. There is neither asphyxia, lipothymia, nor cyanosis: the fits, instead of being developed during the motions of the child, return, on the contrary, when he is in a state of repose.

It would be difficult for me to decide so obscure a question; but it no less follows, from all these observations, that there is still a species of asthma of which percussion and auscultation united may enable us to suspect the material cause during life, before dissection reveals it.

I stop here in the enumeration of the thoracic affections regarded as capable of producing attacks of asthma; for the others are acute or chronic diseases, having their peculiar symptoms, completely distinct from asthma; and if the anatomical characters have sometimes been observed in asthmatics, there could have been simple concomitance only, but by no means reciprocal dependence.

I have said that angina pectoris had been regarded by most authors, as specially allied to organic affections of the heart; I have already shown how little precise information was furnished by auscultation, for detecting the true nature and seat of these lesions. I should add, that were we enabled to diagnosticate in the most exact manner an organic affection of the heart or the great vessels co-existing with angina pectoris, we still would not for all that have *localised* the seat and determined the cause of the group of symptoms denoted by this word. This very clearly and evidently results from a work entitled: *Nouvelles observations sur la nature et la siége de l'angine de poitrine,* published by M. Gintrac of Bordeaux, in the *Journal de Médicine pratique,* or *Recueil des travaux de la Société de Médicine de Bordeaux* (1836). We there read the history of a man, in whom dissection disclosed four aneurismal dilatations of the sub-sternal portion of the aorta. Now, this man had been subject for many years to attacks of angina pectoris, but had been completely relieved of them ten years before his death, and had not afterwards felt them. It is shown by this most interesting case, that other conditions than an alteration of the aorta are necessary for the production of this painful disease. M. Gintrac thinks that the nerves of the cardiac plexus are probably affected, and that they are the cause of the violent pain complained of by patients. This opinion will certainly be shared by the great majority of practitioners.

There is then in asthma and angina pectoris a nervous element which no one can doubt, and which renders them in some degree independent of the organic lesions with which they may coincide. Does it follow that auscultation and percussion have not rendered an immense service by facilitating the diagnosis of these lesions? Who would dare maintain this? It is incontestable that in employing a direct treatment against dry catarrh or emphysema, by means of kermes mineral, ipecacuanha given in doses sufficient to produce vomiting, medicinal soap associated with squills and sometimes with gum ammoniac, salts with a predominance of alkali, and sometimes oily frictions on the chest; against contraction of the lungs, by means of bleeding and exercise commended by M. Quissac, as excellent in the treatment of this disease; against engorgement of the thymus gland, by venesection frequently repeated; and lastly against every disease of the heart recognised by auscultation, were its nature suspected only, by emissions of blood, baths and digitalis, we will succeed in disengaging the nervous affection from a complication which could but aggravate it, by causing the attacks to return more frequently than they otherwise would. It often happens, indeed, that by combating the intercurrent diseases, we will thereby at the same time relieve the nervous disease, and without there being any necessity of employing the special remedies which experience has taught to be the most efficacious in its treatment.

But if a rigid investigation of the pectoral organs, by means of auscultation combined with percussion, does not enable us to recognise any lesion whatsoever of those organs, this favorable result will nevertheless follow from such an examination; that in treating essential asthma by suitable means, we shall be sure of not leaving untreated an affection which, although secondary in appearance, would nevertheless countervail the action of narcotics or other remedies directed against the nervous affection: for we may be certain, that the diminution of the attacks will take place only after the organic affection, whatever it may be, shall have been relieved; and that this could not be until after the latter had been recognised, is perfectly plain.

### CHAPTER VI.—RECAPITULATION OF THE PRECEDING CHAPTERS: ARTIFICIAL AUSCULTATION.

After having considered in succession all the diseases of the lungs and heart, and detailed all that diagnosis, and consequently therapeutics, owe to auscultation, it seems to me natural to bring together in one view, by a summary and brief recapitulation, all the improvements which the science of diagnosis has made since the admirable discovery of Laennec. Such a review can but redound still more to the glory of that powerful mind which has created a new symptomatology for so numerous a class of diseases, and thus rendered their diagnosis as simple and easy as before it was obscure and confused. This will be entering still more into the spirit of the programme of this *concours;* for it seems to me, in proposing it to the zeal and emulation of the young physicians of France, the Bordeaux Medical Society wished that there should result a monument to the glory of Laennec, recounting alike the importance of his immense labors and the gratitude with which all practitioners of all times should preserve his memory.

From the exposition which I have given, it results that the immediate consequences of auscultation combined with percussion are the following:

1. A distinction for ever established between pleurisy and pneumonia, diseases which Laennec has shown to differ as much in their physical signs as in their anatomical characters.

2. The diagnosis of pleuritic effusions based on signs so exact, that henceforth any error is impossible: an inestimable service rendered to surgeons, who, previously, possessed so few means for detecting cases of empyema, that in most instances they doubted their diagnosis until the issue of pus from the chest.

3. The diagnosis of pleurisy, established at its onset, rendered independent of the general signs of reaction: so that, to the attentive practitioner, there can no longer be latent pleurisy, any more than latent pneumonia, for what we say of one of these diseases is equally applicable to the other.

4. The discovery of lobular pneumonia, which without auscultation would never have been made: for either the patient gets well, and then nothing reveals the origin of the hæmoptysis which was the sole symptom; or death takes place, and then it is only subsequent to the extension of the phlegmasia to the whole lung, which causes the disappearance of that singular state of the organ to which the name of lobular pneumonia has been given.

5. The discovery of hypostatic pneumonia or pneumonia of the dying; a disease which pathological anatomy had already pointed out, but which had been considered a cadaveric phenomenon. Auscultation has enabled us to detect it during life, and has thereby furnished indications to prevent, and sometimes to relieve it.

6. A differential diagnosis irrevocably established between pulmonary catarrh and phthisis, diseases which are confounded, so to speak, by their general and almost by local symptoms

(except, however, those furnished by auscultation), and which the greater number of practitioners until then regarded as two stages of the same affection, differing only more or less.

7. The establishment of a strong proof against the generation of phthisis by chronic pneumonia, by showing that these two diseases, in the generality of cases, commence inversely, the one at the summit, the other at the base of the lung.

8. The diagnosis of pulmonary cavities resulting from the softening of tubercles, established on such precise and sure data, that not only can we determine their number and extent, but likewise indicate in a certain manner whether they are completely empty, whether they contain pus alone, or air and pus both at once, whether they are deeply situated in the parenchyma, or near the surface of the lungs, etc.

9. The demonstration, until then never furnished in so evident a manner, of the possibility of the cicatrisation of pulmonary caverns, by the approximation of their walls or their reduction to the state of fistulæ. Until that time patients who had escaped from diseases of the chest, were believed not to have had phthisis; which is equivalent to saying that the ancients were not sure of their diagnosis until they saw it confirmed by dissection.

10. A certain diagnosis established in the case of pulmonary emphysema, œdema of the lungs, and dilatation of the bronchiæ: lesions which pathological anatomy had been content to ascertain, or rather had suffered to pass almost unperceived, so far were pathologists from thinking to detect them during life.

11. A diagnosis created for pneumo-thorax; an affection which until then had been regarded as a cadaveric lesion only. Signs furnished for ascertaining whether it is complicated with perforation of the lung, or whether there is still no communication of the cavity of the pleura with the bronchiæ.

12. The facility of following step by step, and with the finger and the eye, if I may say so, the daily progress of acute and chronic diseases of the lungs, in their period of increment as well as in their declension: an inappreciable advantage, which no other method had ever furnished, and which permits us to judge from day to day whether the medication employed should be continued or modified.

13. The discovery of the existence of latent catarrhs, in most cases of continued fever: a fact which had never been, and without auscultation, could not have been noticed.

14. The power of recognising, during life, the organic affections of the lungs and heart which may coexist with nervous diseases, such as asthma and angina pectoris: a knowledge which may lead to the means calculated to relieve, if not to cure those diseases.

In view of such results, who would dare oppose the eulogies which they ought justly to excite, by alleging that auscultation has to this day failed in all the trials which have been made to obtain by it pathognomonic signs of equal certainty for diseases of the heart? True, it has failed, but yet not completely; for auscultation of the heart has discovered sounds in this organ, different from its normal and physiological sounds, and which all are agreed to regard as morbid. Although we may not agree on their intrinsic worth, as signs representing such or such a lesion, they are not the less valuable, inasmuch as their combination with other signs which the patient may present, enables us, by induction, to suspect organic alterations of whose existence it was formerly morally impossible to have any knowledge, other than by dissection. Furthermore, I have stated the source of this uncertainty in the results of auscultation of the heart; I have proved that the want of an unanimously adopted theory for the normal sounds of that organ, was the sole cause of the difficulty experienced in suitably interpreting the normal sounds. Should we then despair of one day seeing this difficulty overcome? And after all, though this consideration may be of moderate value, and I by no means wish it to be inferred from it that we ought to stop here in these inquiries, and not endeavor to solve this last part of the problem of locating the diseases of the thorax by means of auscultation; after all, I say, the organic lesions of the heart are, perhaps, of all diseases, those whose precise location, when detected during life, will furnish the fewest new therapeutic indications to the practioner entrusted with their management. For, up to this day, the therapeutics of the heart have presented, and perhaps will not present for a long time to come, but these two indications: 1. to diminish the mass of blood by general or local bleedings and a suitable dietetic regimen; 2. to moderate the movements of that organ, by means of digitalis and hypnotics; to which may be added this one: to combat the accidental symptoms in proportion as they may present themselves.

It may be said confidently, and without fear of contradiction, that no one before Laennec had made such immense and unquestionable improvement in the art of diagnosis: no one had settled conflicting opinions on so great a number of diseases, had so irrevocably decided such a number of questions until then regarded as incapable of solution; no one had so completely renewed the history of an entire class of diseases as obscure as those of the chest; and all this Laennec has done through his stethoscope; all this has been the consequence of a single idea, that of listening to the sounds of respiration, and of studying the modifications impressed upon them by the numerous and varied diseases which may alter this function. So true is it that genius does not so much consist in a happy conception, as in knowing how to deduce from it all the consequences which may be drawn.

It was a deep conviction of the indispensable necessity for young physicians to possess a perfect knowledge of this art, which suggested to M. Pétrequin, chief surgeon of the Hotel-Dieu at Lyons, the idea of teaching it to students by the method which he calls *artificial auscultation*. This method, which he has developed in a memoir presented to the Academy of Sciences, the 23d January, 1837, and since published in the *Annales de la Société des Sciences médicales et naturelles de Bruxelles* (1838), consists in first exploring detached lungs, sometimes healthy, sometimes diseased, which he inflates by imitating the respiratory motion, which permits the healthy and morbid sounds to be heard. As to the tubal râles, M. Pétrequin obtains them by throwing various injections into the bronchiæ.

Starting from these data, M. Pétrequin then practices auscultation on the dead body, and assures us that he has succeeded in perceiving the different bronchial and pulmonary sounds, the amphoric respiration, etc.; and thus to have been able to diagnosticate pneumonia, hydro-thorax, cavities from phthisis, and perforation of the lung, upon subjects of whose history he was ignorant. Lastly, to obtain artificial auscultation of the voice, M. Pétrequin applies the widened extremity of the stethoscope on the larynx of a person speaking in a loud tone, and the other end of the cylinder upon the origin of the bronchiæ of the subject ausculted, and the immediate result of this ingenious process is the production of cough and of voice in the brocho-pulmonary cavities.

This theory of artificial auscultation is developed in the memoir which I am analysing, with the spirit and talent remarkable in all the productions of M. Pétrequin, and deserves to be favorably received. It is certainly a happy idea to have mechanically reproduced upon the dead body the circumstances which during life give rise to this or that sound, and I know that these artificial sounds bear the greatest resemblance to those which they are intended to represent. Artificial auscultation may then assist very much in training students to this difficult art, of distinguishing normal or pathological sounds which occur in the chest; but can it completely supply the place of the auscultation of patients? I think not. It is, in fact, on the patient only that the student will learn the connection which exists between the physical signs obtained by the aid of the stethoscope, and the other local and general symptoms, which the dead subject cannot present: such as those drawn from the state of the respiration, the nature of the expectoration, the fever, etc. Let us not deceive ourselves; the art of auscultation does not consist only in hearing the morbid sounds well, but also in properly interpreting them; and this interpretation will be so much the more exact and near the truth, the more it accords with all the other symptoms. Now I admit the inconvenience that sometimes occurs, of submitting patients extremely ill, those affected with pneumonia, for example, to the observation of a large number of students, who, not knowing as yet how to auscult, fatigue them much more than a more practised physician. But it is the duty of a prudent and wise clinical professor, to reconcile the interest of the patient with the advancement of the student: a thing that is easily done, and obtains every where.

As to the advantage which artificial auscultation might have of enabling the student to learn this art much quicker than by the ordinary method, which, according to M. Pétrequin would require one year of assiduous attention in a hospital to acquire the skill necessary to prevent error, I confess that it does not seem to me of very great importance; for, admitting that a whole year would be required to attain a sufficient degree of skill in the art of auscultation, is this time too long, compared with the importance of such a study? Besides, this year would not be exclusively devoted to this subject, but would in reality be a year of clinical instruction, during which the student would learn to recognise and to treat, not only the diseases whose diagnosis is obtained by auscultation, but also all others. The problem of medical education does not consist in abridging the duration of study, and causing the candidate to pass rapidly through the series of examinations which is to conduct him to the doctorate, but on the contrary, in making him spend most profitably the years which the legislature has wisely ordained as the duration of this species of novitiate, in order that when this time has expired, he shall present himself for the final trial with sufficient instruction to serve as a guaranty to the society which is about to grant him such great powers.

### CHAPTER VII.—OF AUSCULTATION APPLIED TO CASES FOREIGN TO DISEASES OF THE CHEST.

It was natural to expect, that, as soon as the beautiful discovery of Laennec became known, a crowd of imitators would follow in his steps and endeavor to discover, by the aid of the stethoscope, every sound capable of being produced in our economy. Laennec himself was led to make researches with this object in view; but all these trials were not equally successful, and there are some indeed of which I shall say nothing, such as those which concern abscesses of the liver and the tympanum, the diagnosis of which Laennec had thought to render more easy by this method, which experience has shown to be impracticable. I shall glance rapidly at the other applications which have been made by other authors and in various cases.

1. *Auscultation of fractures.*—M. Lisfranc is the first, and I believe the only one who has made experiments on this subject. He published them in a memoir inserted in the *Archives générales de Médecine* for August, 1823. There was nothing very surprising that the application of the stethoscope should cause the crepitation of the fragments to be more easily and distinctly heard, since this noise generally reaches the naked ear, and is numbered among the signs of fractures by all authors on surgery. But what characterises the work of M. Lisfranc, is the determining of the distance from the fractured point at which the stethoscope would enable the crepitation to be heard. Now, he has found that the sound is propagated to points quite distant from the place of its production, and indeed, in certain cases of fracture of the femur, he has heard it by placing the stethoscope on the cranium.

M. Lisfranc pretends to recognise, by the difference of the sounds heard, whether the fracture be oblique or transverse; if there are several spiculæ, whether there be a liquid extravasated around them. Laennec, who sought to verify the experiments of M. Lisfranc, avers that he has distinguished, by the aid of his stethoscope, the pointed or obtuse form of the fragments, when the thickness of the soft parts did not permit it to be recognised in a precise manner by the hand. In reading such results, which no one has since attained, we cannot help thinking that these authors have somewhat exaggerated them, by a natural tendency of the mind to augment the importance of a new idea on which great hopes are founded.

These hopes have not been realised; by common consent, they are here stationary; and, really, there are few cases where the able surgeon cannot detect a fracture by the ordinary means of investigation; and in difficult cases, where the diagnosis is doubtful, in spite of the most skilful exploration I do not think that the information furnished by the stethoscope would be sufficient to dispel all doubt, and hence its application is useless.

II. *Auscultation of the bladder.*—The object of catheterism practised for the detection of a vesical calculus, is to render sensible to the ear and to the finger conducting the sound, the shock of this instrument against the stone; it is only on this two-fold condition that we can declare the existence of stone in the bladder. But very often this double sensation is doubtful, and the examples, unfortunately still too frequent, of patients cut without having stone, is a sad but convincing proof of it. Laennec has proposed in these ambiguous cases to apply a stethoscope on the pubis, whilst another person explores the bladder with a sound. But still in this case, it is possible that the sound produced by this shock may not reach the stethoscope, and consequently the ear of the observer. It is to obviate this inconvenience, a necessary consequence of the interruption which exists between the place where the sound is produced, and the conductor which should bring this sound to the ear, that M. M. Moreau of St. Ludger and Béhier, have thought to make the ivory plate of the stethoscope, on which the ear is placed, communicate with the flat extremity of the sound by a metallic wire, by means of which the noise is directly transmitted from the extremity of the sound which percusses the stone, to the ear of the observer. These gentlemen have published the essays made by them on this subject, in the *Journal des connaissances médico-chirurgicales*, of 15th April, 1836, and in the *Lancette française* of 7th May, the same year.

M. Leroy d' Etiolles, finding this rigid conductor very inconvenient, inasmuch as the movements which we are obliged to impress on the sound constantly displace it, has modified this instrument in a very ingenious manner, by replacing this conductor by a spiral spring covered with caoutchouc, which renders it flexible, and consequently susceptible of yielding to all the necessary movements, without injuring the transmission of the sounds, the perception of which it must render more easy. It was on the 31st July, 1837, that M. Leroy d' Etiolles presented his new instrument to the Academy of Sciences. This process, added to the numerous ameliorations which lithotrity has affected in the methods of catheterism, will certainly contribute to render errors of diagnosis more and and more infrequent, if it do not altogether prevent them.

III. *Auscultation of aneurisms.*—The auscultation of aneurisms was a natural consequence of that of the heart; but the information which it affords in these diseases has not a very great value, and can by no means elucidate a doubtful diagnosis. Indeed, in cases of aneurism, where any thing is heard, it is a rasping or bellows sound, resulting from the entrance of the blood into the aneurismal sac. But this sound has no scientific value; it proves that the circulation is impeded, and that the wave of blood which presents itself does not find an aperture sufficient to pass beyond; but the compression of an artery by a superincumbent tumor would produce the same effect. Now, the sign which auscultation ought to furnish, would justly be a differential sign, serving to distinguish an aneurismal tumor from a tumor foreign to the artery. I will say almost as much of the peculiar noise designated by the name of *susurrus*, which is sometimes heard in false consecutive aneurisms, and has been attributed to the passage of the blood through the narrow opening which establishes a communication between the cavity of the artery and that of the aneurismal sac. This *susurral* sound likewise shows nothing more than an impediment to the circulation, and the passage of blood through a too narrow space. But in order that it should be really significant, this impediment to the circulation must not depend on any other cause than the aneurism; and we have just seen the contrary.

IV. *Auscultation of the brain.*—The idea of applying the stethoscope upon the cranium, and of thus obtaining pathognomonic signs in diseases of the encephalon, did not occur to any one before Dr. Fisher called the attention of practitioners to this point, in a note read in 1833 before the Boston Society for Medical Improvement, and inserted in the *Medical Magazine,* No. 15, an analysis of which is given by the *Gazette medicale de Paris* of 14th January, 1834. Dr. Fisher has obtained by this process a phenomenon not yet noticed, and which consists in a bellows sound which has always appeared to him to coincide with compression of the brain. By analysing the six observations which he has annexed to his paper, we see indeed that two of the patients on whom he observed the encephalic bellows sound were affected with acute hydrocephalus, two with chronic hydrocephalus, the fifth labored under the effects of a fall from a second story, and the sixth suffered with an organic disease of the brain. In all it was ascertained that the bellows sound, heard in the region of the encephalon, did not proceed from the heart, since it was not heard in the præcordial region. Three patients having got well, it was found that the bellows sound decreased in proportion as the disease diminished in intensity, and disappeared when the convalescence was complete.

Dr. Fisher, in some reflections which are remarkably just, then makes the following deductions: 1. that this sound takes place in the arteries of the brain; for it is synchronous with the pulsations of the temporals and carotids, and the compression of these vessels causes it to cease; 2. that it occurs in the arteries of the base of the cranium, because it is in that place that the somewhat voluminous arteries are found, and where only they are so situated as to be compressed between the cerebral mass and the bones forming the base of the encephalic cavity; 3. and lastly, that it always accompanies compression of the brain.

As to this last conclusion, more than six observations are necessary to establish it. But the diagnosis of encephalic diseases is so obscure, that the indication of a new sign capable of improving it, is indeed an immense service rendered to science. Time and further inquiry will show what we are to expect from this new application of auscultation.

V. *Auscultation of the abdomen.*—Dr. Bright, physician to

Guy's hospital, at London, first pointed out a *sound like new leather* (*bruit de cuir neuf*), which is perceived by the touch and by the ear, in examining the abdomen of certain patients affected with chronic peritonitis. Dr. Corrigan has confirmed these remarks, in an article inserted in the *Dublin Journal of Medical and Chemical Sciences* (1836). But whilst Dr. Bright attributes it to the rubbing of the adhesions established between the two expansions of the peritoneum, Dr. Corrigan thinks, on the contrary, that it ought to cease as soon as these adhesions are formed, and that the only condition capable of producing it, is the deposite of a certain quantity of lymph, moderately thick and consistent, on the surface of the peritoneum. So soon as this lymph grows thick, in order to be organised into false membranes, this sound diminishes, to disappear completely when the adhesions are perfect.

The observations of these two celebrated English practitioners require confirmation; they would furnish a valuable sign to judge of the progress and state of so grave a disease, the diagnosis of which is not always as easy as one might think.

VI. *Auscultation of the sheaths of the muscles and tendons of the fore-arm.*—M. Lalesque of La Teste (Gironde) has inserted in the *Bulletin médical de Bordeaux,* for 1836, a case of rheumatic inflammation of the muscles of the fore-arm, which was accompanied by a rubbing perceptible to the hand which grasped the affected wrist, and by a creaking sound (*bruit de cuir neuf*) which was heard at a distance, and which the ear, applied to the fore-arm, perceived to be very loud. The observation of M. Lalesque, which is extremely curious, is wanting in some details necessary for knowing where this creaking sound took place. The author appears to think that it was in the cellular sheaths which separate the fleshy fasciculi of the muscles of this region; and that the sound which he heard was only the crepitation perceptible by the ear and by the finger, pointed out by MM. Rognetta, Velpeau and Maingault in inflammation of these tendinous sheaths. (Memoir of M. Rognetta, in *Gazette médicale de Paris,* 1834, p. 596).

VII. *Auscultation during pregnancy.*—It remains for me to speak of the most happy application which has ever been made of auscultation: I mean its application to the study of pregnancy.

It does not enter into my plan to discuss whether this idea was original with M. Kergaradec, or whether he borrowed it from others: his priority has nevertheless been contested. Thus it has been said that M. Mayor, of Lausanne, had long previously had the idea of applying the ear upon the abdomen of the mother, to ascertain whether the child was living; which he determined by hearing the pulsations of the fœtal heart. which were very easily distinguished from the pulse of the mother. (*Rapport fait à l Institut,* on Laennec's work *Auscultation médiate*, by Percy). But M. Mayor did not extend his researches farther. It has also been said that Fodéré had somewhere spoken of it; but to M. Kergaradec will always belong the honor of having first called the attention of accoucheurs to this important point in obstetrical practice.

The stethoscope, applied on the abdominal parietes of a pregnant woman, permits two sounds to be heard: one, somewhat similar to the ticking of a watch wrapped in many cloths, is the *sound of the fœtal heart;* the other is a true bellows sound, which M. Kagaradec has designated under the name of *simple pulsation with blowing* (*battement simple avec souffle*) or *placental sound,* because he supposed this sound to occur in the placenta or in that part of the womb where it is attached. Let us study these two sounds separately.

All accoucheurs are unanimously agreed that the first sound is really owing to the pulsations of the fœtal heart. These pulsations, always double like those of the adult heart, are only much more rapid, and their frequency is commonly twice that of the pulse of the mother. Professor Paul Dubois, in an excellent memoir on this subject, read before the Academy of Medicine, November, 1831, has found the average frequency of the pulsations of the fœtal heart, observed on three hundred pregnant women at the *Maternité* of Paris, to be one hundred and forty or one hundred and fifty pulsations per minute. M. Nœgelé, the younger, in his work entitled *Auscultation obstetricale*, published in 1838, has found the average to be only one hundred and thirty-five pulsations per minute, and his researches were made on nearly six hundred women. Moreover, MM. Paul Dubois and Nœgelé agree in affirming that these pulsations present no remarkable difference of frequency, at whatever period of pregnancy they may be explored. M. Nœgelé does not like the comparison which has been made with the *tic-tac* of a watch, and he cannot give a better idea of the first sound than by comparing it to that of the heart of an infant just born. Accordingly he always makes his students commence by the auscultation of new-born infants, in order to accustom them to recognise the fœtal pulsations when auscultíng the mother: and, from the perfect resemblance of these last sounds to those of the heart of a new-born child, M. Nœgelé draws one of his strongest arguments to prove that they are really those of the fœtal heart, and that they can not be attributed to any other cause.

All authors likewise agree in saying that before the fifth month it is not possible to hear the pulsations of the fœtal heart. M. Nœgelé, however, pretends to have discovered, by means of the stethoscope, sounds produced by the motion of the fœtus in the amniotic liquid, several weeks before the woman herself felt the first movements of her child.

The place where the double pulsation is heard, necessarily varies with the position of the child: however, it is not necessary that its dorsal region should be in direct relation with the point of the abdominal parietes on which the stethoscope reposes. Had this opinion, which was that of M. Kergaradec, been true, it would have enabled us by an easy induction, to determine the relations of the pelvis of the mother, without the necessity of touching per vaginam. But it follows from the researches of M. Paul Dubois, that it is sufficient for one of the points of the thorax of the fœtus to be in relation with the abdominal wall; it does not even appear necessary that the stethoscope should be placed immediately over the point of contact, for this author has remarked that the double pulsation is always heard in a radius of some inches around the point where it presents the greatest force. This is more directly shown by an experiment made by Dr. Hoefft, and published in an article of a German journal, under the title of:

*Remarques sur l'auscultation des femmes encientes, faites pendant les années* 1835 *et* 1836 *dans l'Institut impérial de la Maternité à St. Petersbourg;* which I find analysed in the *Gazette Mèdicale de Paris,* 1838 (p. 808). M. Hoefft enclosed in a small jar hermetically sealed, a watch surrounded by cotton; he then placed the small jar in another vessel full of water which he surrounded with a cloth. By varying the position of the jar in the larger vessel, he discovered that the power of hearing the tic-tac of the watch was modified by the greater or less conductibility of the body transmitting the sound: for, when the watch was near one of the sides of the vessel, it was heard equally well, by applying the stethoscope to one or the other of the extremities of the latter; whilst, when the watch was near the centre of the fluid, it was heard much less distinctly.

If all accoucheurs have been unanimous in admitting the cause of this double sound in the pulsations of the fœtal heart, they have been far from agreeing upon the interpretation which should be given to the second, noticed by M. Kergaradec under the name of the *placental sound,* because he regards it as indicating the situation of the placenta. This is also Laennec's opinion, who, however, does not think it produced by the placenta itself, but by the arterial branch serving principally for the nutrition of the placenta. Laennec founds his opinion on the experiment of Dr. Ollivry of Quimper, who has four times assured himself, by introducing the hand into the womb immediately after the exit of the child, that the point where he had heard the pulsations with a blowing sound before accouchment, corresponded exactly with the attachment of the placenta; and M. Ollivry adds that, if a new proof were necessary for the support of this opinion, it would be found in this fact: that *the blowing sound ceases at the very instant when the umbilical cord is cut.* (Laennec, op. cit., vol. ii, p. 464).

But further researches by MM. Paul Dubois and Nœgelé have shown that the opinion of Laennec and M. Kergaradec is erroneous, and that what these authors have designated under the name of *placental sound,* takes place in the vessels of the uterus, and ought to be called *uterine blowing* (Paul Dubois), *uterine sound* (Nœgelé). Indeed this uterine sound is subject to frequent change of place, and to be heard sometimes in one spot and sometimes in another, or else in two places at once: the placenta, therefore, is not concerned in its production. This uterine sound is nevertheless of great importance, because, being heard long before the pulsations of the fœtal heart, and as soon as the womb is sufficiently developed to reach the level of the pubis, it may serve to make us suspect the existence of a product of conception in the uterus, if, however, it be not shown that the development of the uterus by any tumor whatsoever, may not occasion the production of a similar blowing sound.

Be this as it may, it nevertheless follows from what has just been said, that auscultation is a valuable means of recognising the life of the fœtus in its mother's womb; the existence of the double pulsation is an incontestable proof of it. M. Nœgelé even lays down the inverse proposition as a principle, and thinks that the non-perception of the double pulsation is a sign of the death of the fœtus. But obstetrical auscultation is yet surrounded with too many difficulties, for one to be always sure, that the sound which is not perceived does not exist; it may be that it does not reach the ear on account of something that eludes observation—this is the only conclusion that can be drawn from its absence.

It was hoped that we might be able, by means of auscultation, to detect multiple pregnancy, and to suspect this kind of pregnancy every time that the double fœtal pulsation was heard at two opposite points of the abdomen. It appears from the Clinical Review of the Obstetrical Institute of Paris for the year 1830–31, by Professor Lovati, that in a woman who presented this double pulsation at the fundus of the uterus on the left, and in the right iliac region at the same time, a double pregnancy was diagnosticated on this single sign, and at the delivery they were astonished to see with what precision the stethoscope had indicated not only the number of fœtuses, but also the position of each of them: for one presented the thighs in the *fourth position* (left sacro-posterior); the other, the occiput in the *second position* (right occipito-anterior). But, in the end, these double pulsations in divers points of the uterus were heard in so many women whose pregnancy was simple, that the uncertainty of this sign had to be admitted. However, M. Paul Dubois thinks that during labor, and when the rupture of the membranes has permitted the waters to escape, and consequently leaves no chance for the fœtus to change its position, the presence of two children may be discovered; but then the two double pulsations must be heard simultaneously, situated in two opposite points, and consequently the one stronger than the other. M. Paul Dubois, who has sometimes heard them in this case, has always found them synchronous with each other, and thinks that they can be thus simultaneonsly heard only after the rupture of the two amniotic sacs, or at least of one of them.

The discovery of M. Kergaradec has been fruitful in important results. We feel of what interest it is to ascertain the life of the fœtus, before deciding on this or that obstetrical operation. Thus, to give an example, let us suppose a case of procidentia of the cord: when it shall have been replaced in the uterus, how are we to ascertain the success of this maneuvre? the integrity of the double pulsations of the fœtal heart will be the only certain sign, and if it be ascertained, the natural termination of the labor may be awaited, whilst the progressive feebleness of these pulsations would prove the contrary and justify the intervention of art.

I ought, however, in conclusion, to recall the wise precept of M. Paul Dubois, who does not wish that the accoucheur should seek solely in the state of the fœtal circulation reasons for acting or for waiting, because being independent of the brain, it could not often indicate to us the nearly fatal effects already produced on this organ by the protraction, the difficulties, or the accidents of labor. Furthermore, as M. Nœgelé says, "auscultation is not intended to take the place of exploration by touching; it is a valuable auxiliary added to the other means of investigation, and in conjunction with them must furnish to accoucheurs the most certain rules of conduct."

